# Selenophosphate synthetase 1 deficiency exacerbates osteoarthritis by dysregulating redox homeostasis

**DOI:** 10.1038/s41467-022-28385-7

**Published:** 2022-02-09

**Authors:** Donghyun Kang, Jeeyeon Lee, Jisu Jung, Bradley A. Carlson, Moon Jong Chang, Chong Bum Chang, Seung-Baik Kang, Byung Cheon Lee, Vadim N. Gladyshev, Dolph L. Hatfield, Byeong Jae Lee, Jin-Hong Kim

**Affiliations:** 1 Center for RNA Research, Institute for Basic Science, Seoul, 08826 South Korea; 2 Department of Biological Sciences, College of Natural Sciences, Seoul National University, Seoul, 08826 South Korea; 3 Mouse Cancer Genetics Program, National Cancer Institute, National Institutes of Health, Bethesda, MD 20892 USA; 4 Department of Orthopaedic Surgery, Seoul National University College of Medicine, Boramae Hospital, Seoul, 07061 South Korea; 5 Department of Orthopaedic Surgery, Seoul National University Bundang Hospital, Seongnam, 13620 South Korea; 6 Department of Biotechnology, College of Life Sciences and Biotechnology, Korea University, Seoul, 02841 South Korea; 7 Division of Genetics, Department of Medicine, Brigham and Women’s Hospital and Harvard Medical School, Boston, MA 02115 USA; 8 Interdisciplinary Program in Bioinformatics, Seoul National University, Seoul, 08826 South Korea

**Keywords:** Senescence, Translational research, Osteoarthritis

## Abstract

Aging and mechanical overload are prominent risk factors for osteoarthritis (OA), which lead to an imbalance in redox homeostasis. The resulting state of oxidative stress drives the pathological transition of chondrocytes during OA development. However, the specific molecular pathways involved in disrupting chondrocyte redox homeostasis remain unclear. Here, we show that selenophosphate synthetase 1 (SEPHS1) expression is downregulated in human and mouse OA cartilage. SEPHS1 downregulation impairs the cellular capacity to synthesize a class of selenoproteins with oxidoreductase functions in chondrocytes, thereby elevating the level of reactive oxygen species (ROS) and facilitating chondrocyte senescence. Cartilage-specific *Sephs1* knockout in adult mice causes aging-associated OA, and augments post-traumatic OA, which is rescued by supplementation of N-acetylcysteine (NAC). Selenium-deficient feeding and *Sephs1* knockout have synergistic effects in exacerbating OA pathogenesis in mice. Therefore, we propose that SEPHS1 is an essential regulator of selenium metabolism and redox homeostasis, and its dysregulation governs the progression of OA.

## Abstract (web summary)

Osteoarthritis is caused by the gradual accumulation of oxidative stress in cartilage. Here, the authors show that dysregulation of the selenium metabolic pathway underlies a shift in redox homeostasis in chondrocytes, leading to chronic osteoarthritic changes in joints.

## Introduction

OA is a leading cause of disability, imposing a large socioeconomic burden, the incidence of which increases with age and mechanical joint overload. OA is primarily characterized by cartilage destruction but also involves other pathological changes at the whole-joint level, including synovial inflammation, osteophyte formation, and subchondral bone sclerosis^1–3^. Cartilage homeostasis is maintained by chondrocytes, a major resident cell type of cartilage^4^. Accumulating evidence indicates that imbalance in redox status resulting in oxidative stress in chondrocytes is a crucial event that disturbs cartilage homeostasis during OA development^5–10^.

Selenium is an essential nutrient and trace element that is of vital importance to redox homeostasis, which exerts its physiological role through selenoproteins that contain selenocysteine (Sec) at the active site^11,12^. Selenophosphate is the selenium donor used to synthesize Sec that is co-translationally incorporated into selenoproteins by Sec tRNA^[Ser]Sec^ that decodes an in-frame UGA codon^11,12^. Two paralogs of selenophosphate synthetases (SEPHSs), SEPHS1 and SEPHS2, have been identified in mammals. SEPHS2 forms a complex with SEPHS1 and Sec synthase (SEPSECS)^13,14^, which in turn catalyzes the formation of Sec tRNA^[Ser]Sec^.

There has been growing interest in the potential significance of the selenium metabolic pathway in the pathogenesis of OA, considering the role of selenoproteins in redox regulation^15^ and the detrimental effects of oxidative stress in OA development^7–10^. The association of selenium metabolism with OA pathogenesis has been generally recognized in the context of the protective role of selenium as a nutritional supplement in various epidemiological studies^16,17^. In addition, selenoprotein gene polymorphisms are associated with increased susceptibility to OA development^18–20^. However, the precise mechanism by which the selenium metabolic pathway is dysregulated during OA and its contribution to the pathological transition of chondrocytes has remained elusive to date. Here, we demonstrate that SEPHS1 is an essential regulator of selenium metabolism, whose deficiency limits the synthesis of stress-related selenoproteins and disrupts redox homeostasis in chondrocytes. The dysregulated selenium metabolic pathway triggers oxidative damage and induces chondrocyte senescence, thereby accelerating degenerative processes of the cartilage matrix during OA pathogenesis.

## Results

### SEPHS1 is downregulated in the osteoarthritic cartilage of human and mice

**Fig. 1:**
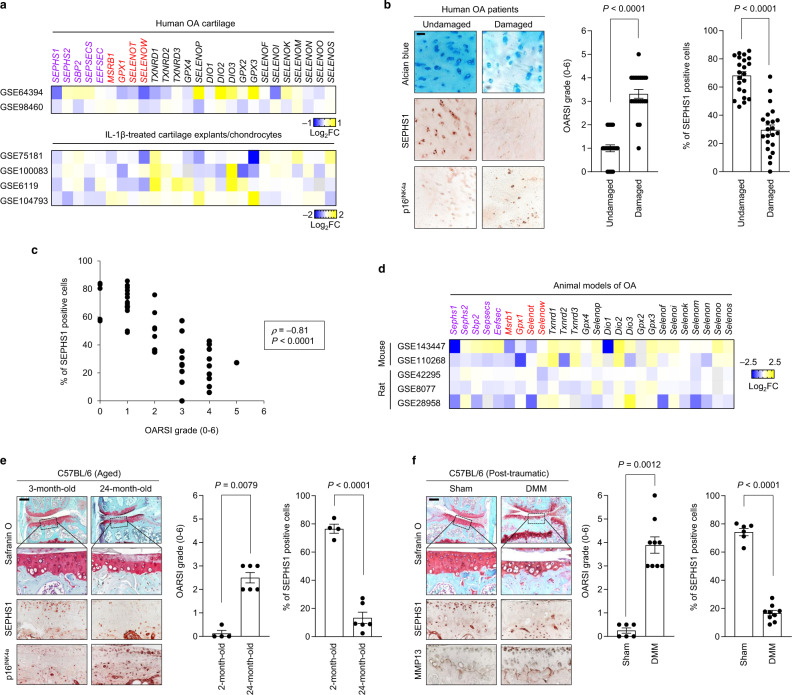
SEPHS1 expression is downregulated in the human and mouse OA cartilage. **a** Fold change (FC) heatmaps of the expression of components of selenium metabolic pathways (purple), stress-related selenoproteins (red), and other selenoproteins (black) in human OA and OA-relevant conditions. Public transcriptome datasets generated from human OA cartilage (GSE64394 and GSE98460), IL-1β-treated cartilage explant (GSE100083), and IL-1β-treated chondrocytes (GSE75181, GSE6119, and GSE104793) were analyzed. **b** Cartilage sections from the undamaged or damaged region of human OA cartilage were stained with Alcian blue and immunostained against SEPHS1 and p16^INK4a^. The extent of cartilage destruction was evaluated by the OARSI grading system and SEPHS1-positive cells were quantified (*n* = 22). **c** Spearman’s rank correlation between OARSI grade and SEPHS1 positivity using human knee cartilage samples from (**b**). **d** FC heatmaps of the expression of the indicated genes in the cartilage of OA animal models. Public transcriptome datasets generated from OA models of mice (GSE143447 and GSE110268) and rats (GSE42295, GSE8077, and GSE28958) were analyzed. **e** Safranin O staining of cartilage sections and immunohistochemistry of SEPHS1 and p16^INK4a^ from 3- and 24-month-old mice. The extent of cartilage destruction was evaluated by the OARSI grading system and SEPHS1-positive cells were quantified (*n* = 4, 6 respectively). The inset in the images is shown as magnified images in the bottom row. **f** Safranin O staining of cartilage sections and immunohistochemistry of SEPHS1 and MMP13 from control (sham) and post-traumatic OA cartilage (8 weeks after DMM) of mice. The extent of cartilage destruction was evaluated by the OARSI grading system and SEPHS1-positive cells were quantified (*n* = 6, 9 respectively). The inset in the images is shown as magnified images in the bottom row. Scale bars: **b** 50 μm, **e**, **f** 200 μm. **b**, **e**, **f** Data represent means ± s.e.m. *P* values are from two-tailed Mann–Whitney *U* test (**b**, **e**, **f**; middle panel), two-tailed *t* test (**b**, **e**, **f**; right panel), or two-tailed Spearman’s rank correlation test (**c**). Cohen’s *d* effect sizes are provided in Supplementary Table 8.

We sought to identify a regulator of the selenium metabolic pathway in chondrocytes with specifically altered expression in OA conditions. Toward this end, we extensively analyzed the transcriptome data available through the Gene Expression Omnibus (GEO) database, focusing on the components of selenium metabolic pathways (*SEPHS1*, *SEPHS2*, *SBP2*, *SEPSECS*, and *EEFSEC*), along with selenoproteins *(MSRB1*, *GPX1*, *SELENOT*, *SELENOW*, *TXNRD1*, *TXNRD2*, *TXNRD3*, *GPX4*, *SELENOP*, *DIO1*, *DIO2*, *DIO3*, *GPX2*, *GPX3*, *SELENOF*, *SELENOI*, *SELENOK*, *SELENOM*, *SELENON*, *SELENOO*, and *SELENOS*). Among the candidate regulators of selenium metabolic pathways, the expression of *SEPHS1* was downregulated in two independent human OA transcriptome datasets (Fig. 1a). Similarly, *SEPHS1* expression was consistently downregulated in IL-1β-treated cartilage explants and chondrocytes (Fig. 1a). Overall, the expression of stress-related selenoproteins (*MSRB1*, *GPX1*, *SELENOT*, and *SELENOW*) was downregulated in these transcriptome datasets. The expression of SEPHS1 protein was also markedly downregulated in human OA cartilage (Fig. 1b, c). SEPHS1 was robustly expressed in undamaged regions of the arthritic cartilage but was barely detectable in the OA-affected regions of human cartilage (Fig. 1b). The expression of p16^INK4a^, a biomarker of cellular senescence^21^, was specifically increased in OA-damaged cartilage. Moreover, the SEPHS1 positivity showed a strong negative correlation with OARSI grade based on Spearman’s rank correlation coefficient (*ρ* = −0.81, *P* = 6.14 × 10^−19^; Fig. 1c).

Similarly, *Sephs1* transcripts were decreased in various animal models of OA, along with reduced expression of stress-related selenoprotein genes (Fig. 1d). The expression of SEPHS1 protein was markedly suppressed in an aged mouse model of knee OA whereas the expression of p16^INK4a^ was increased in the aging-associated OA cartilage (Fig. 1e). We also used the destabilization of the medial meniscus (DMM) surgery as a mouse model of post-traumatic OA^22^. After the surgical induction of OA, SEPHS1 expression was substantially decreased while the expression of MMP13, a key catabolic enzyme involved in osteoarthritic cartilage degradation^23^, was upregulated (Fig. 1f).

### SEPHS1 deficiency causes the dysregulation of redox homeostasis and promotes senescence in chondrocytes

**Fig. 2:**
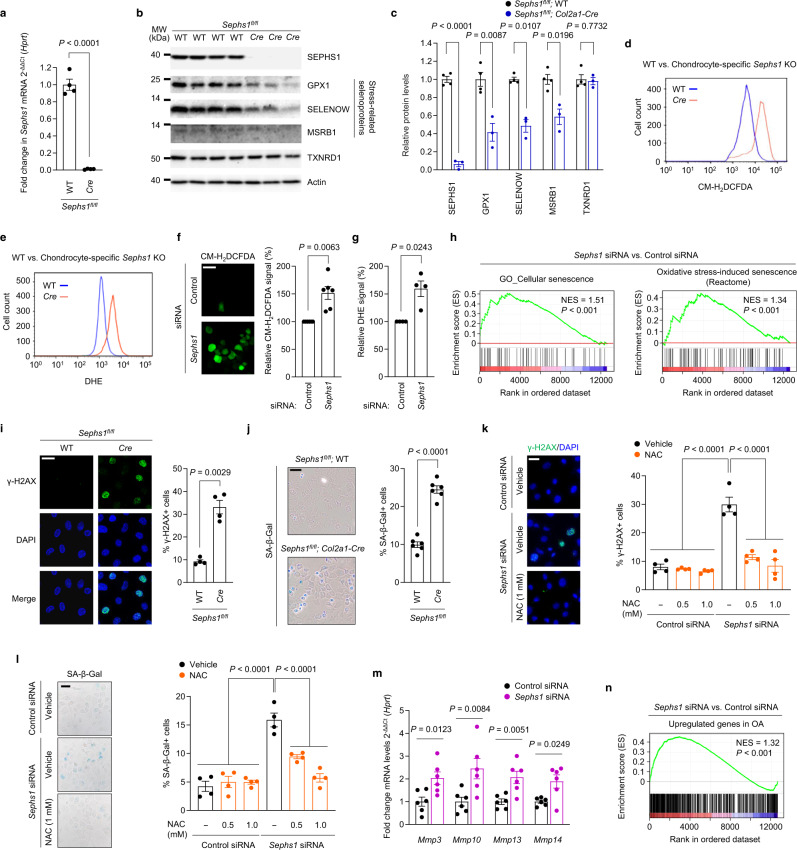
Downregulation of SEPHS1 in OA cartilage leads to oxidative stress-induced cellular senescence in chondrocytes. **a** Relative mRNA expression level of *Sephs1* in primary cultured chondrocytes isolated from *Sephs1*^*fl/fl*^ or *Sephs1*^*fl/fl*^*; Col2a1-Cre* mice (*n* = 4). **b** Western blot analysis of selenoproteins in primary cultured chondrocytes isolated from *Sephs1*^*fl/fl*^ (*n* = 4) and *Sephs1*^*fl/fl*^*; Col2a1-Cre* mice (*n* = 3). **c** Quantification of protein levels in (**b**) (*n* = 4, 3 respectively). **d**, **e** Fluorescence-activated cell sorting (FACS) analysis of **d** CM-H_2_DCFDA and **e** DHE fluorescence in primary cultured chondrocytes isolated from *Sephs1*^*fl/fl*^ and *Sephs1*^*fl/fl*^*; Col2a1-Cre* mice. **f**, **g** Immunofluorescence staining and quantification of **f** CM-H_2_DCFDA (*n* = 6) and **g** DHE (*n* = 4) fluorescence in chondrocytes transfected with negative control siRNA or siRNA targeting *Sephs1*. **h** GSEA of ‘Cellular senescence’ and ‘Oxidative stress-induced senescence’ gene sets in chondrocytes transfected with negative control siRNA or siRNA targeting *Sephs1*. **i** Immunofluorescence staining of γ-H2AX and quantification of γ-H2AX positivity in primary cultured chondrocytes isolated from *Sephs1*^*fl/fl*^ and *Sephs1*^*fl/fl*^*; Col2a1-Cre* mice (*n* = 4). **j** SA-β-Gal staining and quantification of SA-β-Gal positivity in primary cultured chondrocytes isolated from *Sephs1*^*fl/fl*^ and *Sephs1*^*fl/fl*^*; Col2a1-Cre* mice (*n* = 6). **k**, **l** Quantification of **k** immunofluorescence positivity of γ-H2AX and **l** SA-β-Gal positivity in primary cultured chondrocytes transfected with negative control siRNA or siRNA targeting *Sephs1* followed by NAC treatment at the indicated doses (*n* = 4). **m** Relative mRNA expression of SASP factors in chondrocytes transfected with negative control siRNA or siRNA targeting *Sephs1* (*n* = 6). **n** GSEA of the ‘Upregulated genes in OA’ gene set in chondrocytes transfected with negative control siRNA or siRNA targeting *Sephs1*. Scale bars: **f**, **i**, **k** 25 μm, **j**, **l** 50 μm. **a**, **c**, **f**, **g**, **i**–**m ** Data represent means ± s.e.m. *P* values are from two-tailed *t* test (**a**, **c**, **f**, **g**, **i**, **j**, **m**) or two-way ANOVA followed by Dunnett’s post-hoc test (**k**, **l**). For GSEA plots in **h**, **n**, enrichment plots are displayed with the determined nominal *P* value and normalized enrichment score (NES). Unprocessed immunoblot images are provided in Supplementary Fig. 10.

The molecular pathogenesis of OA involves attenuated stress response activities in chondrocytes, which ultimately lead to various metabolic stresses, manifested by mitochondrial dysfunction, oxidative stress, and chronic inflammation^6,24^. Therefore, we further explored how SEPHS1 deficiency affects the expression of stress-related selenoproteins, a subclass of selenoproteins synthesized by the Um34 isoform of Sec tRNA^[Ser]Sec^; the non-Um34 isoform supports the synthesis of a subclass of housekeeping selenoproteins^11^. Because homozygous deletion of *Sephs1* in mice (*Sephs1*^–/–^) was embryonic lethal, we evaluated the effect of *Sephs1* knockout in chondrocytes by establishing cartilage-specific conditional knockout (CKO) mice (*Sephs1*^*fl/fl*^*; Col2a1-Cre*). *Sephs1* transcripts (Fig. 2a) and SEPHS1 proteins (Fig. 2b, c) were nearly undetectable in primary cultured chondrocytes isolated from *Sephs1*-CKO mice. *Sephs1* knockout in chondrocytes substantially attenuated the expression of stress-related selenoproteins such as glutathione peroxidase 1 (GPX1), selenoprotein W (SELENOW), and methionine sulfoxide reductase B1 (MSRB1), whereas the expression of housekeeping selenoprotein, thioredoxin reductase 1 (TXNRD1) was unaffected (Fig. 2b, c).

Since the stress-related selenoproteins downregulated by SEPHS1 deficiency have oxidoreductase functions, we next examined the impact of *Sephs1* knockout on ROS levels in chondrocytes. Chondrocytes isolated from *Sephs1*-CKO mice exhibited a significantly elevated level of ROS as determined by two redox-sensitive fluorescent indicators: CM-H_2_DCFDA and dihydroethidium (DHE) that measure levels of intracellular H_2_O_2_ and O_2_^•−^, respectively (Fig. 2d, e and Supplementary Fig. 1). Similarly, small interfering RNA (siRNA)-mediated knockdown of *Sephs1* resulted in significant accumulation of ROS in chondrocytes (Fig. 2f, g). To comprehensively elucidate the effect of SEPHS1 deficiency at the whole-transcriptome level, we performed RNA sequencing for chondrocytes treated with control and *Sephs1* siRNAs. Gene ontology (GO) and pathway analysis indicated that the differentially regulated genes by *Sephs1* knockdown were mainly associated with functional annotations related to ‘DNA damage response’, ‘cell cycle arrest’, and ‘oxidative stress response’ (Supplementary Figs. 2 and 3). Notably, these annotations are closely related to senescence^25,26^, a critical cellular event disturbing matrix homeostasis during OA development^24,27^. Indeed, gene set enrichment analysis (GSEA) revealed that genes related to ‘cellular senescence’ and ‘oxidative stress-induced senescence’ were positively enriched in the whole transcriptome obtained from SEPHS1-deficient chondrocytes (Fig. 2h).

To further verify the mechanistic link between SEPHS1 deficiency and cellular senescence, we monitored the accumulation of DNA damage and senescence-associated β-galactosidase (SA-β-gal) activity in response to the loss of SEPHS1. Chondrocytes isolated from conditional *Sephs1* knockout mice exhibited a marked increase in the DNA damage response, as indicated by strong formation of γ-H2AX nuclear foci, and facilitated entry into cellular senescence, as evidenced by increased SA-β-gal activity (Fig. 2i, j). Likewise, chondrocytes treated with *Sephs1* siRNA exhibited substantially higher percentages of γ-H2AX foci and SA-β-gal positivity (Supplementary Fig. 4a, b). We hypothesized that the accumulation of ROS caused by SEPHS1 deficiency is responsible for eliciting persistent DNA damage and cellular senescence in chondrocytes. In support of this hypothesis, treatment of SEPHS1-deficient chondrocytes with NAC, a well-characterized ROS scavenger^28^, abolished the *Sephs1* knockdown-mediated increases in γ-H2AX and SA-β-gal positivity (Fig. 2k, l). Chondrocyte senescence is critically associated with low-grade inflammation and matrix degeneration in OA pathogenesis through secretion of pro-inflammatory cytokines and proteases, which are collectively referred to as senescence-associated secretory phenotype (SASP) factors^24,29^. *Sephs1* knockdown in chondrocytes promoted the expression of various SASP factors, including matrix proteases responsible for the degeneration of the cartilage matrix (Fig. 2m and Supplementary Fig. 4c). Furthermore, GSEA revealed that OA-associated genes upregulated in patients^30,31^ were overall increased in chondrocytes upon *Sephs1* knockdown (Fig. 2n).

### Temporal knockout of SEPHS1 in adult cartilage accelerates aging-associated OA

**Fig. 3:**
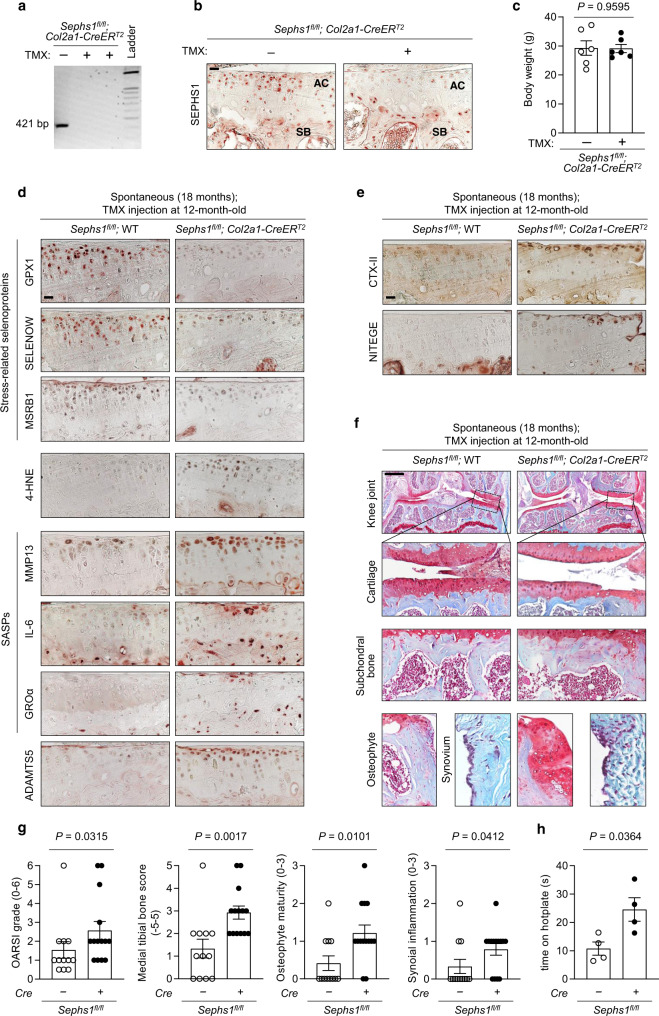
Chondrocyte-specific *Sephs1* knockout in adult mice accelerates aging-associated OA development in knee joints. **a** PCR verification of *Sephs1* inducible conditional knockout (iCKO) after five intraperitoneal injections of tamoxifen (TMX) in 12-week-old *Sephs1*^*fl/fl*^*; Col2a1-CreER*^*T2*^ mice. **b** Immunostaining of SEPHS1 in knee joint sections displaying the articular cartilage (AC) and subchondral bone (SB) of 21-week-old WT and *Sephs1*-iCKO littermates. **c** Body weight measurements at 8 weeks after five times injections of vehicle or TMX in 12-week-old *Sephs1*^*fl/fl*^*; Col2a1-CreER*^*T2*^ mice (*n* = 6). **d**, **e** *Sephs1*^*fl/fl*^ or *Sephs1*^*fl/fl*^*; Col2a1-CreER*^*T2*^ mice were injected with TMX at 12 months of age, and the appearance of aging-associated OA phenotypes was analyzed at 18 months. **d** Stress-related selenoproteins (GPX1, SELENOW, and MSRB1), 4-HNE, SASPs (MMP13, IL-6, and GROα), ADAMTS5, and **e** cartilage matrix neoepitopes (telopeptides of type II collagen, CTX-II and aggrecan neoepitope, NITEGE) were detected by immunohistochemistry in cartilage sections. **f** Joint sections were stained with safranin O, fast green, and hematoxylin. The inset in the images is shown as magnified images in the bottom row. **g** Scores of OA manifestations, including cartilage destruction, subchondral bone sclerosis, osteophyte formation, and synovial inflammation (*n* = 12 for *Sephs1*^*fl/fl*^; *n* = 14 for *Sephs1*^*fl/fl*^*; Col2a1-CreER*^*T2*^). **h** Hotplate pain assay in 18-month-old WT and *Sephs1*-iCKO mice (*n* = 4). Scale bars: **b**, **d**, **e** 25 μm, **f** 500 μm. **c**, **g**, **h** Data represent means ± s.e.m. *P* values are from two-tailed *t* test (**c**, **h**) or two-tailed Mann–Whitney *U* test (**g**). Cohen’s *d* effect sizes are provided in Supplementary Table 8. Mankin scores and SBP thickness measurements are provided in Supplementary Figs. 11 and 12.

Collectively, these results indicated that SEPHS1 downregulation impairs the cellular oxidoreductase capacity of chondrocytes, resulting in elevated ROS levels. The resulting state of oxidative stress causes persistent DNA damage, driving chondrocytes into a cellular senescence state, thereby eliciting OA-associated transcription and secretion patterns. To investigate the role of SEPHS1 deficiency in OA development in vivo, we aimed to establish a cartilage-specific *Sephs1* knockout mouse model displaying normal skeletal development. However, the *Sephs1*-CKO mice obtained by crossing *Col2a1-Cre* mice with *Sephs1*^*fl/fl*^ mice exhibited growth retardation at postnatal day 5.5 compared to their control littermates (Supplementary Fig. 5). Therefore, we used a tamoxifen-inducible system to temporally knock out *Sephs1* in skeletally mature, adult mice. We verified cartilage-specific *Sephs1* deletion in the knee joint of these *Sephs1*-inducible CKO mice (iCKO) after tamoxifen injection (Fig. 3a–c).

We then examined how the cartilage-specific deletion of *Sephs1* in adult mice affects the pathogenesis of aging-associated OA. Aged (18-month-old) *Sephs1*-iCKO mice showed decreased levels of stress-related selenoproteins, including GPX1, SELENOW, and MSRB1 (Fig. 3d). At the same time, they exhibited an increased level of 4-hydroxynonenal (4-HNE) compared with that of age-matched control mice, indicating dysregulation of redox homeostasis in the articular cartilage (Fig. 3d). The expression of senescence-associated pro-inflammatory cytokines such as IL-6 and GROα was upregulated in *Sephs1*-iCKO mice. The expression levels of MMP13 and ADAMTS5, two crucial effectors of OA cartilage destruction, were also increased in the cartilage of *Sephs1*-iCKO mice (Fig. 3d), coinciding with the increased production of type II collagen telopeptide CTX-II and the aggrecan neoepitope NITEGE (Fig. 3e). Consistent with these molecular-level changes, aged *Sephs1*-iCKO mice exhibited significant spontaneous cartilage destruction compared with age-matched wild-type (WT) mice. Other OA manifestations, including subchondral bone sclerosis, osteophyte development, and synovitis, were augmented in *Sephs1*-iCKO mice, indicating accelerated progression of aging-associated OA (Fig. 3f, g). Furthermore, aged *Sephs1*-iCKO mice showed an increased response time in hotplate analysis as a behavioral test of sensory dysfunction^29,32,33^ as compared with control mice (Fig. 3h), demonstrating that SEPHS1 loss exacerbates sensory impairment that is common during OA progression and a main cause of chronic disability in OA patients^34^.

### SEPHS1 deficiency aggravates post-traumatic OA development

**Fig. 4:**
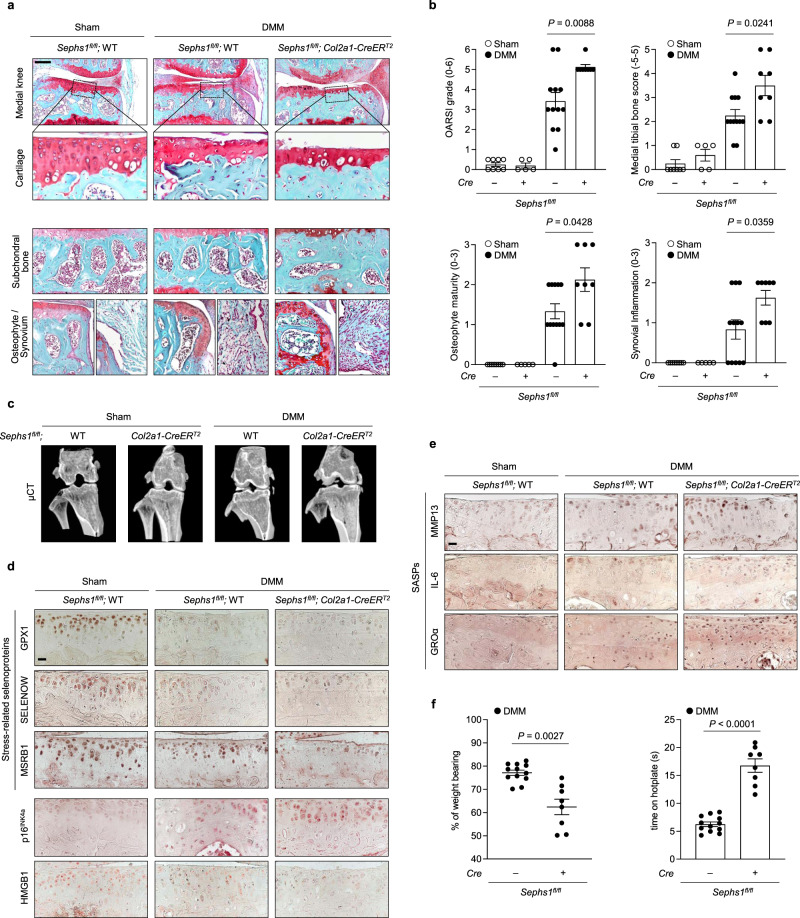
Chondrocyte-specific temporal *Sephs1* knockout exacerbates post-traumatic OA in mice. **a** *Sephs1*^*fl/fl*^ or *Sephs1*^*fl/fl*^*; Col2a1-CreER*^*T2*^ 12-week-old mice were injected with TMX five times and subjected to sham operation or DMM surgery. Joint sections were stained with safranin O, fast green, and hematoxylin. The inset in the images is shown as magnified images in the bottom row. **b** Cartilage destruction, subchondral bone sclerosis, osteophyte formation, and synovial inflammation determined by safranin O/hematoxylin staining and scored (*n* = 8 for sham-operated WT; *n* = 5 for sham-operated *Sephs1*-iCKO; *n* = 12 for DMM-operated WT; *n* = 8 for DMM-operated *Sephs1*-iCKO). **c** Representative microcomputed tomography (μCT) images of sham- or DMM-operated WT and *Sephs1*-iCKO mice. **d** Stress-related selenoproteins (GPX1, SELENOW, and MSRB1), p16^INK4a^, HMGB1, and **e** SASPs (MMP13, IL-6, and GROα) were detected by immunohistochemistry in cartilage sections. **f** Hotplate pain assays in DMM-operated WT and *Sephs1*-iCKO mice (left panel, *n* = 12 for WT; *n* = 8 for *Sephs1*-iCKO). The percentage of weight placed on the sham- or DMM-operated limb versus the contralateral limb of WT and *Sephs1*-iCKO mice (right panel, *n* = 12 for WT; *n* = 8 for *Sephs1*-iCKO). Scale bars: **a** 200 μm, **d**, **e** 25 μm. **b**, **f** Data represent means ± s.e.m. *P* values are from Kruskal–Wallis test followed by Mann–Whitney *U* test (**b**) or two-tailed *t* test (**f**). Cohen’s *d* effect sizes are provided in Supplementary Table 8. Mankin scores and SBP thickness measurements are provided in Supplementary Figs. 11 and 12.

Genetic deletion of *Sephs1* also augmented DMM-induced, post-traumatic OA development based on histological assessment of cartilage destruction, subchondral bone sclerosis, osteophyte maturation, and synovial inflammation (Fig. 4a, b). Microcomputed tomography (μCT) of the subchondral trabecular bone showed remarkable enhancement in OA-associated bone remodeling following DMM surgery in *Sephs1*-iCKO mice as compared with that of control mice (Fig. 4c). Synovial ectopic calcifications were not observed in the synovium of DMM-operated WT and *Sephs1*-iCKO mice, as verified by the negative staining against type II collagen expression and alkaline phosphatase (ALP) activity (Supplementary Fig. 6a, b). At the molecular level, *Sephs1* knockout in the knee joints of DMM-operated mice impaired the synthesis of stress-related selenoproteins and caused a marked increase of senescent cell populations based on increased immunostaining of p16^INK4a^ and reduced nuclear HMGB1 (Fig. 4d), resulting in increased expression of SASP factors, including MMP13, IL-6, and GROα (Fig. 4e). *Sephs1*-iCKO mice exhibited substantial weight imbalance between the surgically treated (ipsilateral) and untreated (contralateral) legs, indicating augmented pain development in the ipsilateral knee joint^35^. DMM-operated *Sephs1*-iCKO mice showed a longer response time on the hotplate, demonstrating sensory impairments compared to WT mice (Fig. 4f).

### Antioxidant treatment rescues the augmented OA phenotypes in SEPHS1-deficient mice

**Fig. 5:**
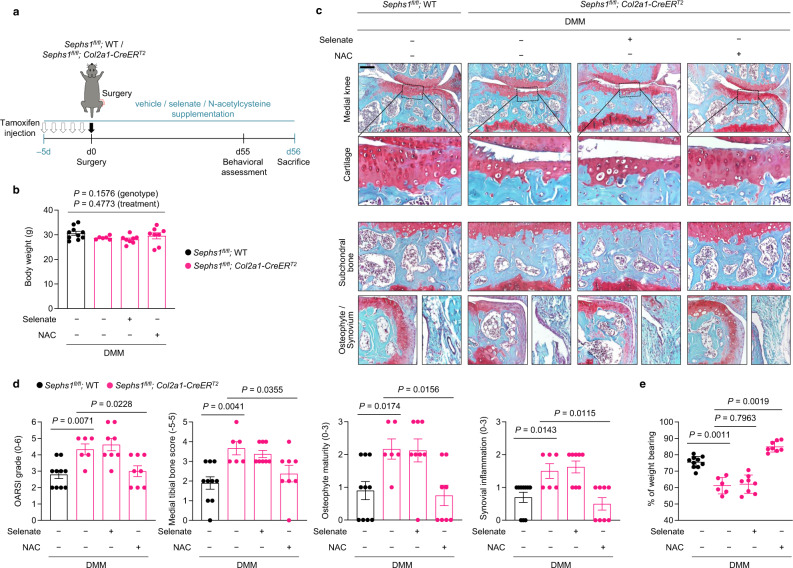
NAC treatment rescues the exacerbated OA phenotypes in *Sephs1*-iCKO mice. **a** Schematic illustration of NAC or dietary selenate supplementation in the post-traumatic OA model of *Sephs1*-iCKO mice. **b** Body weight of 21-week-old DMM-operated mice after completion of the supplementation scheme (*n* = 10 for DMM-operated WT mice treated with vehicle; *n* = 6 for DMM-operated *Sephs1*-iCKO mice treated with vehicle; *n* = 8 for DMM-operated *Sephs1*-iCKO mice supplemented with selenate; *n* = 8 for DMM-operated *Sephs1*-iCKO mice treated with NAC). **c** Joint sections were stained with safranin O, fast green, and hematoxylin. The inset in the images is shown as magnified images in the bottom row. **d** Cartilage destruction, subchondral bone sclerosis, osteophyte formation, and synovial inflammation determined by safranin O/hematoxylin staining and scored (*n* = 10, 6, 8, 8 respectively). **e** The percentage of weight placed on the DMM-operated limb versus the contralateral limb of WT and *Sephs1*-iCKO mice treated with or without NAC or selenate determined using a static weight bearing test (*n* = 10, 6, 8, 8 respectively). Scale bar: **c** 200 μm. **b**, **d**, **e** Data represent means ± s.e.m. *P* values are from two-way ANOVA followed by Tukey’s post hoc test (**b**) or S–R–H test followed by Mann–Whitney *U* test (**d**, **e**). Cohen’s *d* effect sizes are provided in Supplementary Table 8. Mankin scores and SBP thickness measurements are provided in Supplementary Figs. 11 and 12.

To further demonstrate whether increased oxidative stress is responsible for the augmented OA phenotypes observed in DMM-operated *Sephs1*-iCKO mice, we conducted rescue experiments with supplementation of NAC (Fig. 5a). In parallel, we tested whether the supplementation of selenate, an inorganic form of selenium that is widely used in a selenium substitution strategy^36–38^, is sufficient to rescue the genetic deficiency of *Sephs1*. *Sephs1*-iCKO mice were supplemented with NAC, selenate, or vehicle starting at 5 days before DMM surgery; these supplementations did not affect the body weight of the animals as compared with that of vehicle-supplied WT mice over the course of rescue experiments (Fig. 5b). We next examined whether the OA manifestations augmented by *Sephs1* loss were alleviated by supplementation of NAC or selenate. The supplementation of NAC effectively reduced the whole-joint OA manifestations observed in *Sephs1*-iCKO mice (Fig. 5c, d). Consistently, weight distributions between the surgically treated (DMM) and untreated legs were also restored following NAC supplementation (Fig. 5e). In contrast, selenate supplementation did not rescue the OA phenotypes observed in *Sephs1-*iCKO mice. Taken together, increased oxidative stress is responsible for the exacerbation of OA caused by the genetic deficiency of *Sephs1* in mice.

### Deficiency of selenium intake and SEPHS1 synergistically exacerbates OA development

**Fig. 6:**
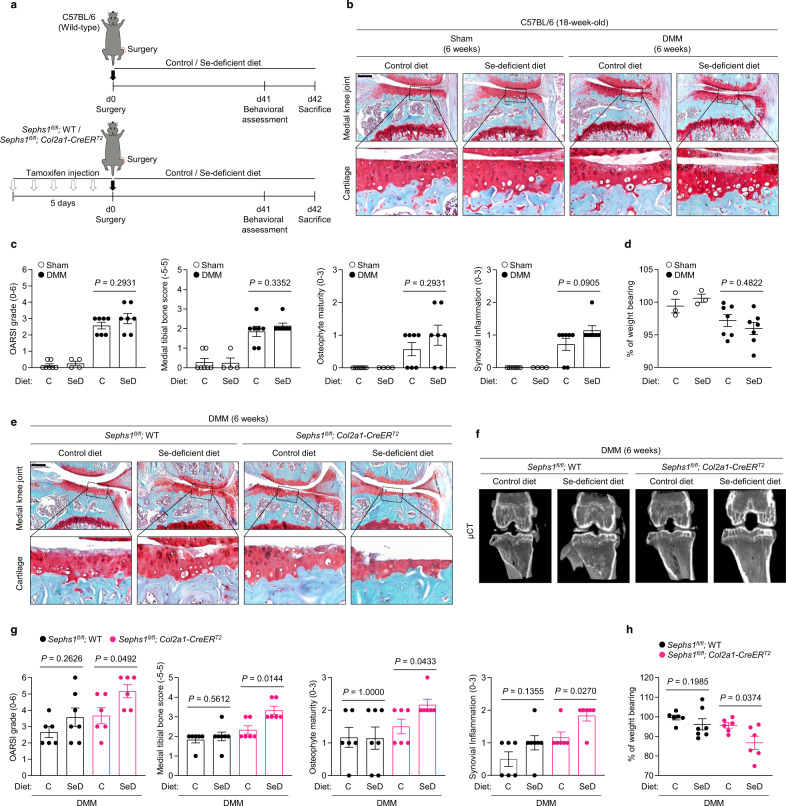
Dietary selenium deficiency augments the progression of OA in *Sephs1*-iCKO mice. **a** Schematic illustration of dietary selenium depletion in the post-traumatic OA model of C57BL/6 mice (top) or *Sephs1*-iCKO mice (bottom). **b** Twelve-week-old C57BL/6 mice received sham operation or DMM surgery. Joint sections were stained with safranin O, fast green, and hematoxylin. The inset in the images is shown as magnified images in the bottom row. **c** Cartilage destruction, subchondral bone sclerosis, osteophyte formation, and synovial inflammation determined by safranin O/hematoxylin staining and scored (*n* = 7 for sham-operated mice fed with control (C) diet; *n* = 4 for sham-operated mice fed with selenium-deficient (SeD) diet; *n* = 7 for DMM-operated mice fed with control diet; *n* = 7 for DMM-operated mice fed with selenium-deficient diet). **d** Percentage of weight placed on the sham- or DMM-operated limb versus the contralateral limb over 15 min analyzed using a dynamic weight bearing test (*n* = 7, 4, 7, 7 respectively). **e** Twelve-week-old WT and *Sephs1*-iCKO mice were operated with sham or DMM surgery. Joint sections were stained with safranin O, fast green, and hematoxylin. The inset in the images is shown as magnified images in the bottom row. **f** Representative μCT images of sham- or DMM-operated WT and *Sephs1*-iCKO mice fed with the indicated diets. **g** Cartilage destruction, subchondral bone sclerosis, osteophyte formation, and synovial inflammation determined by safranin O/hematoxylin staining and scored (*n* = 6 for DMM-operated WT mice fed the control diet; *n* = 7 for DMM-operated WT mice fed the selenium-deficient diet; *n* = 6 for DMM-operated *Sephs1*-iCKO mice fed the control diet; *n* = 6 for DMM-operated *Sephs1*-iCKO mice fed the selenium-deficient diet). **h** Percentage of weight placed on the DMM-operated limb versus the contralateral limb over 15 min analyzed by a dynamic weight bearing test (*n* = 6, 7, 6, 6 respectively). Scale bars: **b**, **e** 200 μm. **c**, **d**, **g**, **h** Data represent means ± s.e.m. *P* values are from Kruskal–Wallis test followed by Mann–Whitney *U* test (**c**, **d**) or S–R–H test followed by Mann–Whitney *U* test (**g**, **h**). Cohen’s *d* effect sizes are provided in Supplementary Table 8. Mankin scores and SBP thickness measurements are provided in Supplementary Figs. 11 and 12.

Finally, we investigated the effects of selenium deficiency^39,40^ on post-traumatic OA (Fig. 6 and Supplementary Fig. 7). There were no significant differences in OA manifestations observed between selenium-deficient (SeD) and control diet groups after DMM surgery (Fig. 6a–c). Similarly, the selenium dietary condition did not cause significant changes in dynamic weight bearing, suggesting that nutritional restriction of selenium in adults is not sufficient to enhance OA progression (Fig. 6d). However, lack of dietary selenium together with SEPHS1 deficiency synergistically accelerated OA progression in the post-traumatic OA model as cartilage destruction, subchondral bone sclerosis, and synovial inflammation were evident as early as 6 weeks post-DMM (Fig. 6e–g and Supplementary Fig. 8a). Consistent with these results, we found significant loss of stress-related selenoprotein expression and upregulation of SASP factors in mice with the combined deficiency of selenium and SEPHS1 (Supplementary Fig. 9). This effect was further validated in pain measurement based on dynamic weight bearing (Fig. 6h and Supplementary Fig. 8b).

## Discussion

The etiology of OA is multifactorial, including mechanical stresses imposed on the joint and predisposition factors such as aging. These OA risk factors have been associated with elevated levels of oxidative stress in the joint tissues^24,41,42^. In fact, accumulation of the oxidative burden is considered a hallmark of chondrocytes undergoing osteoarthritic changes^5–7^. Oxidative stress results from excessive ROS production and the loss of cellular oxidoreductase capacity. Emerging evidence suggests that oxidative stress is mechanistically linked to initiating the pathological changes in chondrocytes through the acquisition of senescent phenotypes^10^. Therefore, it is crucial to elucidate the underlying mechanism that disrupts the redox homeostasis in chondrocytes during OA progression.

**Fig. 7:**
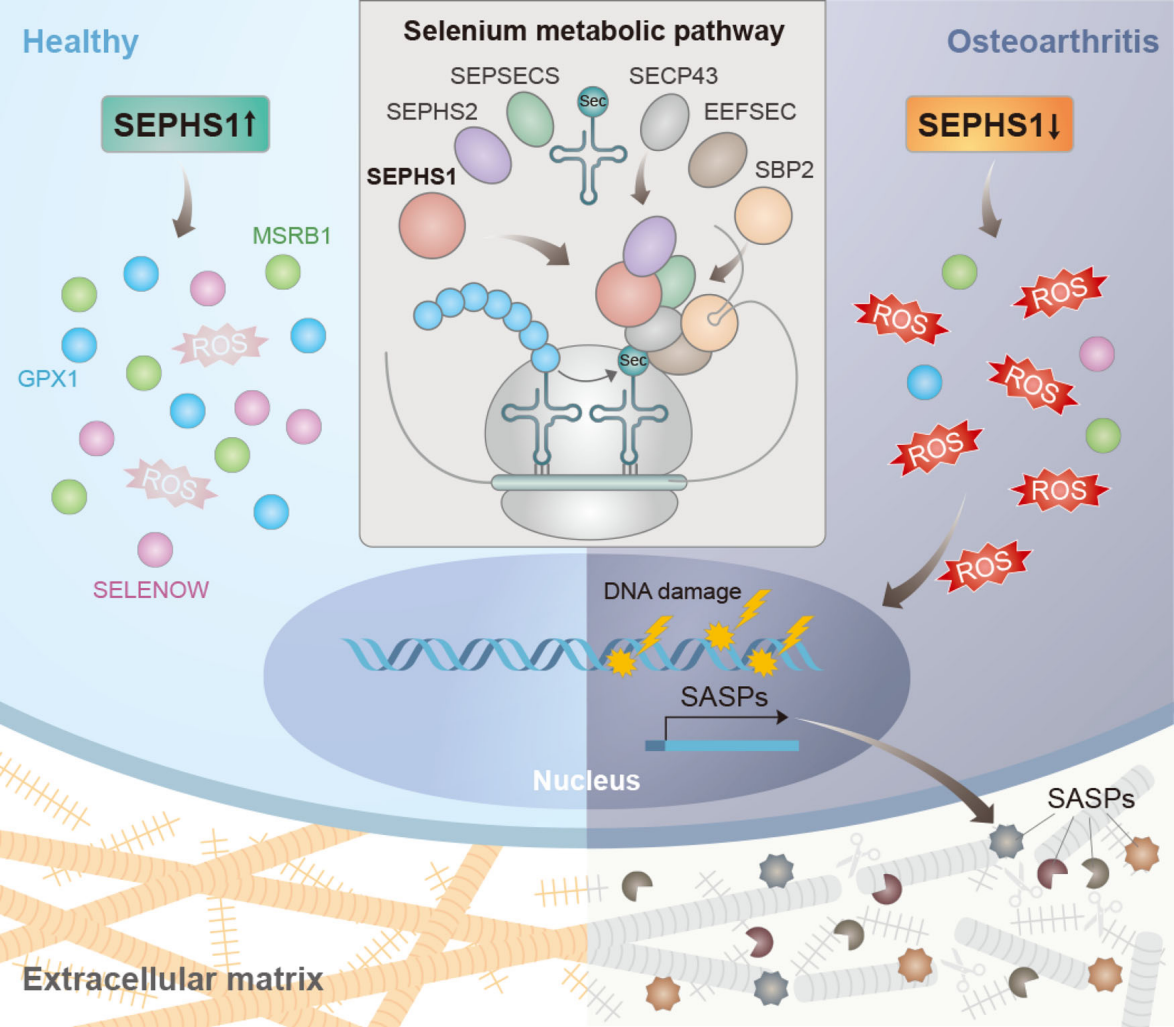
Schematic diagram representing the molecular pathway by which SEPHS1 deficiency exacerbates OA development. SEPHS1 expression is downregulated in OA chondrocytes. SEPHS1 deficiency impairs cellular capacity to synthesize stress-related selenoproteins with oxidoreductase functions in chondrocytes, elevating ROS levels. This event, in turn, enhances DNA damage, cellular senescence, and SASPs expression, causing the catabolic degeneration of the cartilage matrix by fostering chronic inflammation in the joint environments.

In this study, we demonstrate that dysregulation of the selenium metabolic pathway underlies a shift in redox homeostasis in chondrocytes (Fig. 7). Among the various regulators of the selenium metabolic pathway, SEPHS1 expression was found to be markedly downregulated in both the human and mouse OA cartilage. Deficiency in SEPHS1 expression was responsible for the decreased expression of stress-related selenoproteins with oxidoreductase activity, including GPX1, SELENOW, and MSRB1, which are known to rank low on the hierarchy of expression during selenium restriction^43,44^. The reduced oxidoreductase capacity elicited by SEPHS1 deficiency leads to increased intracellular ROS levels and the subsequent onset of cellular senescence in chondrocytes. One of the most distinct features of senescence is the onset of SASPs^45^. Our results indicate that the senescence induced by SEPHS1 deficiency promotes the expression of SASP factors, which in turn mediates the catabolic degeneration of the cartilage matrix and fosters chronic inflammation in the joint environments.

Pre-clinical animal studies indicated an association of selenium deficiency with abnormal skeletal development and growth retardation. In line with these studies, *Col2a1* promoter-driven, chondrocyte-specific deletion of *Trsp*, which encodes Sec tRNA^[Ser]Sec^, caused chondronecrosis and immature cartilage development in mice^46^. Similarly, we observed that chondrocyte-specific knockout of *Sephs1* (*Sephs1*^*fl/fl*^*; Col2a1-Cre*) led to growth retardation, further corroborating the significance of the selenium metabolic pathway in skeletal development. However, OA develops after the skeletal system is fully mature and along with aging. To specifically explore the role of the selenium metabolic pathway in OA development, we temporally knocked out *Sephs1* in the cartilage of skeletally mature adult mice. In the pre-clinical settings of aging-associated and post-traumatic OA, we showed that genetic deletion of *Sephs1* augmented OA phenotypes in terms of histological, radiological, and pain assessments. Selenium-deficient feeding combined with *Sephs1*-iCKO further promoted OA pathogenesis in mice. Therefore, our results reveal that the selenium metabolic pathway plays an essential role in maintaining joint tissue homeostasis beyond its role in the developmental process of the musculoskeletal system.

In this study, we observed that selenium-deficient feeding alone was not sufficient to significantly aggravate post-traumatic OA in mice. To our knowledge, there are a few other intervention studies that examined the effect of selenium restriction on musculoskeletal systems in murine models. In these studies, selenium deficiency induced growth retardation in rats^47^, and caused fibrocartilage formation and ultimate degeneration of the articular cartilage in mice^48^. However, it should be noted that these previous studies were conducted in the context to examine the developmental defects elicited by selenium deficiency. For this purpose, the animals used in these studies were observed after two generations of selenium-deficient feeding. The aim of our study was to specifically explore the pathological effect of selenium deficiency in adults with relevance to OA pathogenesis. Therefore, the mice were fed the selenium-deficient diet only after they were fully grown to the adult stage so as to exclude the possibility that any developmental abnormities caused by selenium depletion would affect the pathogenesis of OA in adult mice.

We propose that SEPHS1 is an essential regulator of the selenium metabolic pathway whose dysregulation disrupts redox homeostasis and governs the pathogenesis of OA. Considering the protective effects of selenoproteins with oxidoreductase capacity in maintaining cartilage homeostasis, strategies aimed at sustaining selenium metabolism may be an effective therapeutic and preventive approach for OA.

## Methods

### Collection of human tissue samples

Human OA cartilage specimens were obtained from OA patients undergoing total knee arthroplasty at SNU Boramae Medical Center. The Institutional Review Board (IRB) of SNU Boramae Medical Center approved the collection of human biological materials (IRB No. 30-2017-48) and the IRB of Seoul National University approved the use of these materials (IRB No. E1803/003-009). Written informed consent was obtained from all subjects before the total knee replacement surgery. Patient information, including sex, age, height, weight, and BMI, is summarized in range in Supplementary Table 1. We used the samples obtained from female patients only. Human cartilage tissue sections were stained with Alcian blue and graded according to the OARSI grading system^49^.

### Mice

For the establishment of the *Sephs1* knockout mice line, floxed *Sephs1* (*Sephs1*^*fl/fl*^) mice were generated^50^ and crossed with *Col2a1-Cre* or *Col2a1-CreER*^*T2*^ mice. For the establishment of the aging-associated spontaneous OA model, 12-month-old male *Sephs1*^*fl/fl*^ or *Sephs1*^*fl/fl*^*; Col2a1-CreER*^*T2*^ mice were intraperitoneally injected with 80 μg/g body weight TMX dissolved in corn oil (Sigma Aldrich) daily for five consecutive days. For the establishment of the post-traumatic OA model, 12-week-old male *Sephs1*^*fl/fl*^ or *Sephs1*^*fl/fl*^*; Col2a1-CreER*^*T2*^ mice were injected with 80 μg/g body weight TMX for five consecutive days. *Cre*-mediated *Sephs1* excision activity in the knee joint cartilage of TMX-injected 12-week-old *Sephs1*^*fl/fl*^*; Col2a1-CreER*^*T2*^ (*Sephs1*-iCKO) mice was validated by PCR using genomic DNA as a template. Tissue Genomic DNA extraction SV mini prep kit (MG MED) was used for extraction of genomic DNA from paraffin-embedded chondrocytes in the knee articular cartilage. The PCR primers used for genotyping are listed in Supplementary Table 2. *Selenop* KO mouse line was obtained from The Jackson Laboratory (#008201).

### Selenium-deficient diet in mice

Selenium dietary regimens for the mice were based on those previously described^40,51–53^. Briefly, 12-week-old male *Sephs1*^*fl/fl*^ or *Sephs1*^*fl/fl*^*; Col2a1-CreER*^*T2*^ mice were injected with 80 μg/g body weight TMX for five consecutive days. Mice were fed an SeD torula yeast-based diet (0.026 μg/g Se) or an adequate selenium control diet (0.4 μg/g Se) beginning 5 days before sham or DMM operation. The mice were fed the same diet for six additional weeks after their surgeries until they were sacrificed.

### Experimental OA in mice

All animal experiments were approved by the SNU Institutional Animal Care and Use Committee (IACUC No. SNU-151202-6, SNU-190919-6, SNU-190910-1, SNU-191115-3). The study complied with all relevant ethical regulations for animal testing and research. The design, analysis, and reporting of animal experiments were performed following the Animals in Research: Reporting of In Vivo Experiments guidelines (ARRIVE; http://www.nc3rs.org.uk/arrive-guidelines). The mice were housed in a specific pathogen-free animal facility at SNU. Animals were maintained under constant temperature (23–25 °C) and humidity (45–65%) with controlled light-dark cycles (12:12 h). Mice were fed standard laboratory chow or the SeD as indicated above *ad libitum*. Selenate supplementation or NAC treatment was performed by supplying mice with drinking water dissolved with sodium selenate (1 ppm)^36,38^ or NAC (1 mg/ml)^9^. *Sephs1*-iCKO mice were supplemented with NAC, selenate, or vehicle beginning 5 days before DMM surgery. Spontaneous induction of aging-associated OA in *Sephs1*-iCKO mice was examined in 18-month-old mice. After five times of intraperitoneal TMX injections in 12-month-old *Sephs1*^*fl/fl*^ or *Sephs1*^*fl/fl*^*; Col2a1-CreER*^*T2*^ mice, the mice were sacrificed at 18 months of age. Post-traumatic OA was induced by DMM surgery^22^ in 12-week-old (WT C57BL/6) or 13-week-old (*Sephs1*^*fl/fl*^ or *Sephs1*^*fl/fl*^*; Col2a1-CreER*^*T2*^) mice; mice operated with sham surgery served as controls. The mice were sacrificed at 6 or 8 weeks after DMM or sham surgery. As an aging-associated OA model in WT mice, 24-month-old C57BL/6 male mice were used for histological analysis, and 3-month-old mice were used as controls. The degree of cartilage destruction in the knee joints was evaluated by safranin O staining and scored using the OARSI grading system^54^.

### Histology and immunohistochemistry

Human OA cartilage specimens were cryoembedded in OCT compound and sectioned (7 μm thickness). OA-affected cartilage samples were acquired from the medial side of the tibial plateau and the relatively undamaged regions from the lateral side of the tibial plateau were used as controls. Prior to histological analysis, the cryosections were air-dried for 20 min and fixed in pre-chilled acetone for 10 min. Human OA cartilage sections were stained with Alcian blue or immunohistochemical staining. Knee joint tissues collected from the murine aging-associated OA model, murine post-traumatic OA model, and their respective controls were fixed with 4% paraformaldehyde (PFA), decalcified in 0.5 M EDTA (pH 7.4), processed by dehydration in an increasing concentration gradient of ethanol, and incubated in xylene. The samples were embedded in paraffin and sectioned (5 μm thickness). For histological staining, the sections were deparaffinized in xylene, hydrated in a decreasing concentration gradient of ethanol, and stained with safranin O or immunohistochemical staining. Comprehensive histological evaluation of whole-joint tissues was conducted by two orthopedic pathologists at SNU Boramae Medical Center with extensive experience in evaluating human and mouse OA. Sections were reviewed by two additional pathologists and approximately 90% complete agreement was achieved for the OARSI grade. The observers were blinded to the genotype, feeding, or surgical condition of the mice, and section images were randomized to avoid observer bias. Cartilage destruction was assessed using safranin O staining and scored using the OARSI grading system (0–6)^54^ and the Mankin scoring system (0–14) (Supplementary Fig. 11)^55^. The medial tibial bone sclerosis (grade –5 to 5) was scored by assessing the subchondral trabecular bone to bone marrow ratio^29^. Subchondral bone plate (SBP) thickness was also measured (Supplementary Fig. 12)^39^. Osteophyte maturity (grade 0–3) was scored by examining the anteromedial tibia^56^. Synovial inflammation was scored on an arbitrary scale (0–3) depending on the infiltration of inflammatory cells into the synovial membrane^57^. Primary antibodies used for immunohistochemistry were as follows: SEPHS1 (Santa Cruz, sc-365945; dilution 1:100), p16^INK4a^ (Proteintech, 10883-1-AP; dilution 1:100), MMP13 (Abcam, ab51072; dilution 1:100), GPX1 (Abcam, ab22604; dilution 1:100), SELENOW (Rockland, 600-401-A29; dilution 1:100), 4-hydroxynonenal (Abcam, ab46545; dilution 1:100), IL-6 (Santa Cruz, sc-130326; dilution 1:100), GROα (R&D systems, MAB453; dilution 1:100), ADAMTS5 (Abcam, ab41037; dilution 1:100), CTX-II (C-telopeptide of type II collagen; Novus, NBP2-59386; dilution 1:100), NITEGE (ADAMTS-cleaved aggrecan neoepitope; MD Bioproducts, 1042003; dilution 1:100), HMGB1 (Abcam, ab18256; dilution 1:100), and type II collagen (Sigma Aldrich, MAB8887; dilution 1:100). A previously developed anti-MSRB1 antibody was used in this study^58^. Secondary antibodies used for immunohistochemistry were as follows: donkey anti-mouse IgG (H&L) conjugated with Biotin-SP (Jackson ImmunoResearch, 715-065-150; dilution 1:200), donkey anti-rabbit IgG (H&L) conjugated with Biotin-SP (Jackson ImmunoResearch, 711-065-152; dilution 1:200), and goat anti-rat IgG (H&L) conjugated with Biotin (Abcam, ab6844; dilution 1:200). ALP activity in knee joints was detected using nitroblue tetrazolium chloride (NBT)/5-bromo-4-chloro-3-indolylphosphate (BCIP) Ready-to-Use Tablets (Roche).

### Dynamic weight bearing

As an indicator of OA-associated pain, the weight distribution placed on the hindlimbs was measured using a dynamic weight bearing system (Bioseb). Animals were placed in a chamber and allowed to move freely at least three times before measurement. A sensor array placed on the floor of the chamber measured the position, surface area of the footprint, and degree of foot pressure, which corresponds to weight. The movement of mice was recorded using a camera placed at the top of the chamber. Data obtained from the sensor array and video were matched to calculate the weight applied on each limb over the dynamic movement for a 15 min period (three times recording for 5 min each). Data were presented as a percentage of the weight placed on the DMM-operated ipsilateral limb versus that on the non-surgical contralateral limb. The observers were blinded to the genotype, feeding, or surgical condition of the mice.

### Static weight bearing

Incapacitance measurements were conducted using the Incapacitance Meter for mice/rats (IITC Life Science, 600MR) one day prior to sacrifice. Mice were trained to walk into and remain in the chamber at least three times before measurement. The adaptation was performed until the mice remained still and did not lean toward either side of the chamber. Before measurement, each hindlimb was positioned on each recording pad. The weight placed on each recording pad was measured over 1 s for at least three independent measurements. Data were presented as a percentage of the weight placed on the DMM-operated ipsilateral limb versus that on the non-surgical contralateral limb. The observers were blinded to the genotype, feeding, or surgical condition of the mice.

### Hotplate pain assay

The mice were placed on the hotplate analgesia meter (Columbus Instruments) at 55 °C and data were analyzed^29,32,33,59^. The latency period for a hindlimb response such as paw shaking, licking, or jumping behaviors was recorded as the response time, one day prior to sacrifice. At least three response times were recorded per mouse. The observers were blinded to the genotype, feeding, or surgical condition of the mice.

### Microcomputed tomography

Anesthetized mice were scanned with an in vivo Micro-CT Scanner (Bruker, Skyscan 1276) one day prior to sacrifice. The sham- or DMM-operated hindlimb was fixed and scanned at a 0.4° angle for a total of 515 scans. Scans were obtained using a 70 kV X-ray source voltage, 57 μA current, and composite X-ray filter of 0.5 mm aluminum, and images were produced with a pixel size of 20 μm. The three-dimensional images were constructed using NRecon software (Bruker).

### Cell culture

For primary culture of mouse articular chondrocytes, the cells were isolated from the femoral condyles and tibial plateaus of 5-day-old ICR mice^60^. Chondrocytes were maintained in Dulbecco’s modified Eagle’s medium (DMEM) supplemented with 10% fetal bovine serum (FBS; Gibco), 100 units/ml penicillin, and 100 μg/ml streptomycin. After two days, the cells were treated as indicated in each experiment. Cultured cells were maintained in a humidified 37 °C, 5% CO_2_, and 3% O_2_ atmosphere. For siRNA transfection, the cells were treated with hyaluronidase type I-S (4 units/ml) for 4 h prior to transfection under serum-free conditions. Transfection was performed using METAFECTENE PRO (Biontex) and 50 nM of siRNA according to the manufacturer’s instructions. After 12 h of transfection, the cells were incubated in fresh medium for 48 h. For efficient and long-term knockdown, transfection was carried out twice. All siRNAs, including negative control siRNA, used for RNA interference in the study were purchased from Bioneer and are listed in Supplementary Table 3.

### Immunoblotting

Primary articular chondrocytes isolated from 5-day-old *Sephs1*^*fl/fl*^ or *Sephs1*^*fl/fl*^*; Col2a1-Cre* mice were maintained in DMEM supplemented with 1% FBS, 100 units/ml penicillin, and 100 μg/ml streptomycin. After three days, the cells were washed twice with phosphate-buffered saline (PBS) and lysed with RIPA buffer containing protease inhibitor (Sigma Aldrich). Next, 15 μg of cell lysates were fractionated by SDS-PAGE and transferred onto a nitrocellulose membrane (GE Healthcare). The membranes were blocked with 3% non-fat milk or 2% bovine serum albumin in Tris-buffered saline containing 0.1% Tween 20 for 1 h and incubated with primary antibodies at 4 °C overnight. After removing unbound antibodies by washing three times, the membranes were incubated with secondary antibodies. Immunoreactive protein bands were detected using an ECL substrate (Thermo Scientific) with iBright FL1000 (Thermo Scientific). Primary antibodies used for immunoblotting were as follows: SEPHS1 (Santa Cruz, sc-365945; dilution 1:1000), GPX1 (Abcam, ab22604; dilution 1:1000), SELENOW (Rockland, 600-401-A29; dilution 1:200), TXNRD1 (Santa Cruz, sc-28321; dilution 1:1000), and actin (Santa Cruz, sc-1615; dilution 1:4000). A previously described anti-MSRB1 antibody (dilution 1:5000) was used^58^. Secondary antibodies used for immunoblotting were as follows: goat anti-rabbit IgG (H&L) conjugated with horseradish peroxidase (HRP) (Jackson ImmunoResearch, 111-035-003; dilution 1:5000), goat anti-mouse IgG+IgM (H&L) conjugated with HRP (Jackson ImmunoResearch, 115-035-044; dilution 1:5000), and donkey anti-goat IgG conjugated with HRP (Santa Cruz, sc-2020; dilution 1:10000). Protein band intensity was quantified by densitometric analysis using ImageJ^61^ and normalized to the corresponding actin bands. Unprocessed immunoblot images are provided in Supplementary Fig. 10.

### ROS detection using DHE and CM-H_2_DCFDA staining

For DHE staining, primary culture of mouse chondrocytes was stained with 1 μM DHE (Thermo Scientific) diluted in DMEM without sodium pyruvate for 15 min. For CM-H_2_DCFDA staining, primary culture of chondrocytes was stained with 5 μM CM-H_2_DCFDA (Thermo Scientific) diluted in DMEM without sodium pyruvate for 30 min. The stained cells were washed three times in PBS, fixed in 4% PFA for 20 min, and mounted in PBS. Cells were assayed by flow cytometry using FACS Canto II (BD Biosciences) and BD FACSDiva software (v8.0) or imaged with EVOS FL Cell Imaging System (Thermo Scientific).

### Immunofluorescence

Primary culture of mouse chondrocytes was fixed in 4% PFA for 15 min, permeabilized with 0.1% Triton X-100 in PBS for 10 min, and blocked with 10% normal goat serum in PBS for 1 h. The cells were incubated in primary antibodies against γ-H2AX (Santa Cruz, sc-517348) and normal mouse IgG (Santa Cruz, sc-2025). The cells were washed three times with PBS and incubated with rabbit anti-mouse IgG+IgM (H&L) conjugated with Alexa Fluor 488 (Jackson ImmunoResearch, 315-485-044). The nuclei were stained with DAPI. Stained cells were mounted with ProLong Gold Antifade (Thermo Scientific) and imaged under EVOS FL Cell Imaging System.

### SA-β-Gal staining

SA-β-Gal staining was performed as previously described^62^. In brief, the cells were washed three times with PBS and fixed with 2% PFA and 0.2% glutaraldehyde for 5 min. Fixed cells were washed and incubated in SA-β-Gal staining solution at 37 °C for 12–16 h. After incubation, the cells were washed twice with PBS, followed by mounting in 70% glycerol solution. Stained cells were imaged using Eclipse Ni-U microscope (Nikon). Total cells and SA-β-Gal-positive cells were counted in three random fields per biological replicate.

### Quantitative reverse transcription polymerase chain reaction (qRT-PCR)

Total RNA was isolated using TRI reagent (Molecular Research Center, Inc.) and reverse-transcribed using EasyScript Reverse Transcriptase (TransGen Biotech). To quantitively analyze mRNA transcript levels, cDNA was amplified by qRT-PCR using Power SYBR Green PCR Master Mix (Thermo Scientific) on StepOnePlus Real-Time PCR System v2.3 (Applied Biosystems). The primers used for qRT-PCR are listed in Supplementary Table 4. For qRT-PCR-based quantification data comparing the effect of various treatments or genotypes, we obtained relative quatification with the ΔΔCt method using *Hprt* as an internal control. For cell-based experiments, each set of primary cultured chondrocytes was considered a biologically independent trial. We then averaged the normalized values from multiple biologically independent trials.

### RNA sequencing

Mouse primary chondrocytes were transfected with 50 nM of negative control siRNA or siRNA targeting *Sephs1* for 12 h and incubated in fresh medium for 48 h. Transfection procedures were conducted twice. Three biological replicates were used for each experimental group. Total RNA was extracted using TRI reagent. One microgram of total RNA was converted into cDNA libraries using the TruSeq Stranded mRNA Sample Prep kit (Illumina). Poly-adenylated RNA was purified using magnetic beads conjugated to oligo-dT primers. Purified mRNA was fragmented and converted into first-strand cDNA using random hexamer primers and reverse transcriptase, with the addition of actinomycin D. Second-strand cDNA was prepared by eliminating the RNA and synthesizing the complementary strand in the presence of dUTP. A single A base was added to the 3′ end to enable the ligation of adaptors containing a single T overhang. The cDNA was then amplified by PCR, while the polymerase stops when it encounters a U base to render the complementary strand as a poor template. Final cDNA libraries were analyzed for size distribution, quantified by qRT-PCR (Kapa Library Quant KIT, Kapa Biosystems) using Agilent 2100 Bioanalyzer, and normalized to 2 nM for sequencing. RNA sequencing was performed using NovaSeq 6000 (Illumina). All RNA sequencing procedures were performed by Macrogen. Reads were preprocessed and aligned to the reference genome *Mus musculus (mm10)* using HISAT v2.1.0^63^. HISAT incorporated two types of indexes for alignment: a global whole-genome index and tens of thousands of small local indexes. The reference genome sequence of *mm10* and annotation data were downloaded from the UCSC genome browser (http://genome.ucsc.edu). Transcript assembly and abundance estimation were performed using StringTie v1.3.4d^64,65^. The aligned reads were assembled into known, novel, and alternative splicing transcripts, and the relative abundance of each transcript was quantified in read counts using StringTie v1.3.4d. For statistical analysis, genes with a read count value of zero at least in one sample were excluded. Filtered data were transformed into log_2_(read count + 1) values and subjected to RLE normalization. Statistical significance of the differential expression was determined using nbinomWaldTest of DESeq2. Differential gene expression between negative control siRNA- and si*Sephs1*-treated chondrocytes was determined using a cutoff *P* value < 0.05.

### Bioinformatics analysis of RNA sequencing data and public datasets

Gene ontology analysis of differentially expressed genes was performed using Enrichr (http://amp.pharm.mssm.edu/Enrichr/). Top-ranked terms from WikiPathways, Kyoto Encyclopedia of Genes and Genomes (KEGG), BioCarta, Panther, and GO were selected by their Enrichr *P* values, which were calculated with Fisher’s exact test. For GSEA, gene lists of RNA sequencing data were ranked based on the fold change values. GSEA software v4.0.3 was conducted in pre-ranked mode, with all default parameters, for the ‘GO_Cellular senescence’ and ‘Oxidative stress-induced senescence’ (Reactome) gene sets. GSEA was also performed with the list of 752 genes significantly upregulated in OA as a gene set representing ‘upregulated genes in OA’^30,31^. The genes used for GSEA are listed in Supplementary Tables 5–7. Transcriptome data for OA or OA-relevant conditions in various in vivo and in vitro models were obtained from the GEO database. Transcriptomes of human OA cartilage (GSE64394 and GSE98460), IL-1β-treated cartilage explants (GSE100083), IL-1β-treated chondrocytes (GSE75181, GSE6119, and GSE104793), and cartilage from various OA animal models (DMM surgery: GSE143447; anterior cruciate ligament transection (ACLT) surgery: GSE110268, GSE42295, and GSE8077; monoiodoacetate (MIA) injection: GSE28958) were analyzed. *GPX6*, *SELENOH*, and *SELENOV* were excluded from our transcriptome analysis. The Sec residue of GPX6 is not evolutionarily conserved in mice and rats, and expression levels of *SELENOH* and *SELENOV* were negligible in cartilage (below the cutoff).

### Statistical analysis

All experiments were conducted on at least three independent biological replicates, including all histology and immunohistochemistry experiments represented as micrographs. For in vitro experiments, comparison of experimental groups was carried out by a parametric test based on two-tailed Student’s *t* test, Welch’s *t* test (for qRT-PCR analyses of *Mmp14* in Fig. 2m and *Igfbp7* in Supplementary Fig. 4c), or two-way analysis of variance (ANOVA) followed by Dunnett’s or Tukey’s post hoc test. For in vivo experiments, each independent trial was conducted using an individual mouse. To determine statistically significant differences, a non-parametric test based on Mann–Whitney *U* test was used. For non-parametric analysis in multigroup comparisons, Kruskal–Wallis test followed by Mann–Whitney *U* test or Scheirer–Ray–Hare (S–R–H) test followed by Mann–Whitney *U* test was used. Data quantified based on ordinal grading systems including the OARSI grade and the Mankin score, and scores for subchondral bone sclerosis, osteophyte maturity, and synovial inflammation, whose data points are not continuous and do not follow a normal distribution, were analyzed using non-parametric statistical methods. Separate statistical analyses were performed for sham and DMM groups such that the surgical condition was not considered a variable (as in cases described in refs. ^9,66,67^). Correlations between SEPHS1 positivity and OARSI grade in human OA cartilage samples were measured using Spearman’s rank correlation coefficient *ρ*. The effect sizes of OA histological parameters were calculated using Cohen’s *d* (huge, *d* > 2; very large, *d* = 1.2–1.99; large, *d* = 0.8–1.19; medium, *d* = 0.5–0.79; small, *d* = 0.2–0.49)^68^ and are provided in Supplementary Table 8. Statistical significance was accepted at *P* < 0.05. Statistical analyses were performed using IBM SPSS Statistics 25 or GraphPad Prism 9.0. All the graphs and heatmaps of log_2_(fold change) were plotted using GraphPad Prism 9.0. Cell cultures and animals were randomly assigned to each experimental group and all samples were evaluated in a blinded manner. The sample size *n* required for each group for animal studies to provide sufficient power was determined based on a power analysis calculation (Eq. 1) and the design of a previous study^39^. No exclusion criteria were included in the study. The additional details of statistical analyses for Figs. 2a, k, l, m,  5b and Supplementary Fig. 4c are provided in Supplementary Note 1.

1 $$n=\frac{2{\sigma }^{2}{({z}_{\frac{\alpha }{2}}+{z}_{\beta })}^{2}}{{D}^{2}}$$

where *D* = ${\hat{\mu }}_{{{{\rm{treatment}}}}}\mbox{-}{\hat{\mu }}_{{{{\rm{control}}}}}$, *σ* = standard deviation, *α* = significance level (0.05), and 1 − *β* = power (0.90).

### Reporting summary

Further information on research design is available in the Nature Research Reporting Summary linked to this article.

## Supplementary information

Supplementary Information

Reporting Summary

## Data Availability

The original RNA-seq data generated in this study have been deposited in the GEO database under accession code GSE179535. Transcriptome data for OA or OA-relevant conditions in various in vivo and in vitro models referenced during the study are available in the GEO database (http://www.ncbi.nlm.nih.gov/geo). Transcriptomes of human OA cartilage (GSE64394 and GSE98460), IL-1β-treated cartilage explants (GSE100083), IL-1β-treated chondrocytes (GSE75181, GSE6119, and GSE104793), and cartilage from various OA animal models (DMM surgery: GSE143447; ACLT surgery: GSE110268, GSE42295, and GSE8077; MIA injection: GSE28958) were analyzed. The list of genes significantly upregulated in OA was obtained from previous studies^30,31^. All other relevant data supporting the findings of this study are available within the article and its Supplementary Information file. Source data are provided with this paper.

## Source data

Source Data
