# Supplementary material for: Selenophosphate synthetase 1 deficiency exacerbates osteoarthritis by dysregulating redox homeostasis
Source: Nat Commun. 2022 Feb 9;13:779. doi: 10.1038/s41467-022-28385-7 (PMC8828855; doi:10.1038/s41467-022-28385-7)
Supplement: Supplementary file 1 — Supplementary Information [file 41467_2022_28385_MOESM1_ESM.pdf]

SUPPLEMENTARY INFORMATION

Selenophosphate synthetase 1 deficiency exacerbates osteoarthritis by dysregulating redox homeostasis

Kang et al.

Supplementary Figures 1–12

Supplementary Tables 1–8

Supplementary Note 1

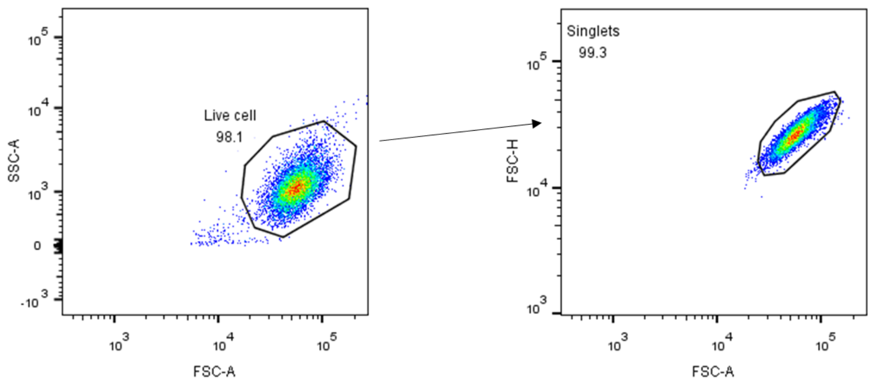

Supplementary Fig. 1 Exemplified gating strategy for flow cytometry analysis in Fig. 2d,e. Mouse chondrocytes were gated by forward scatter (FSC) and side scatter (SSC) area (A) according to cell size and granularity to get rid of any debris or large clumps. Then, singlet cells were gated using FSC-A and FSC-height (H).

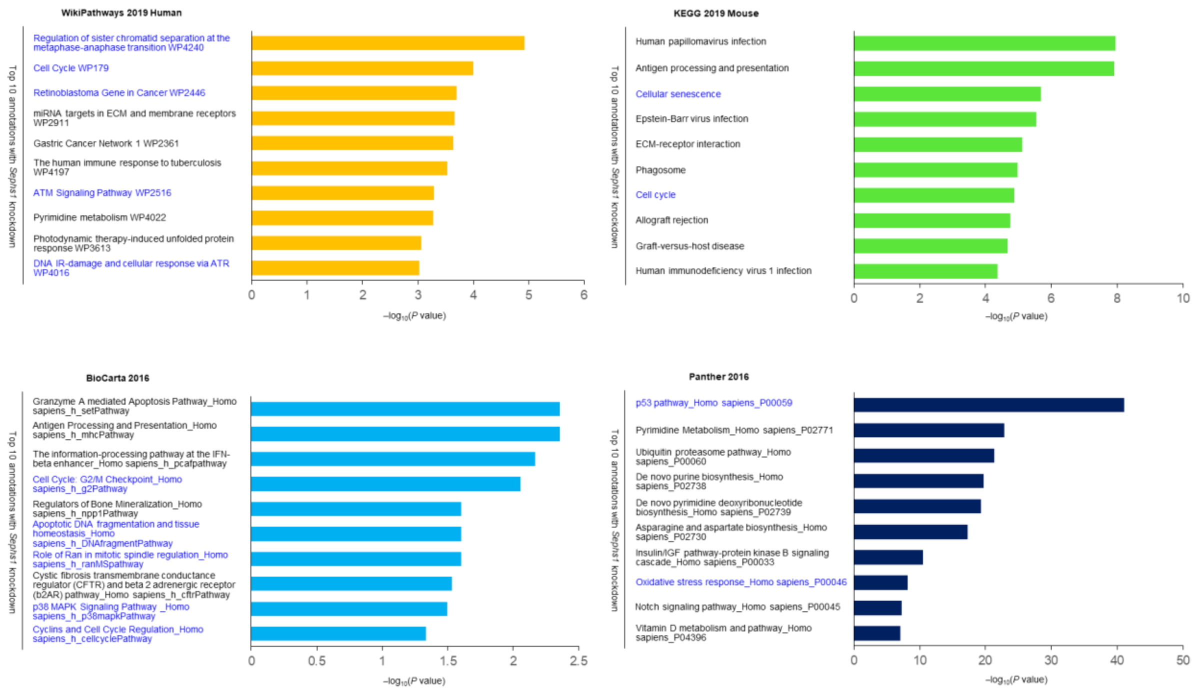

Supplementary Fig. 2 Transcriptome analysis reveals that loss of SEPHS1 activates pathways related to DNA damage response, cell cycle regulation, and cellular senescence in chondrocytes. Top 10 annotations of differentially expressed genes following knockdown of *Sephs1* in chondrocytes from Enrichr Pathways analysis, based on WikiPathways 2019 Human, KEGG 2019 Mouse, Biocarta 2016, and Panther 2016 pathway databases; the corresponding $-\log_{10}$(*P* values) are presented as graphs. *P* values are from Fisher's exact test.

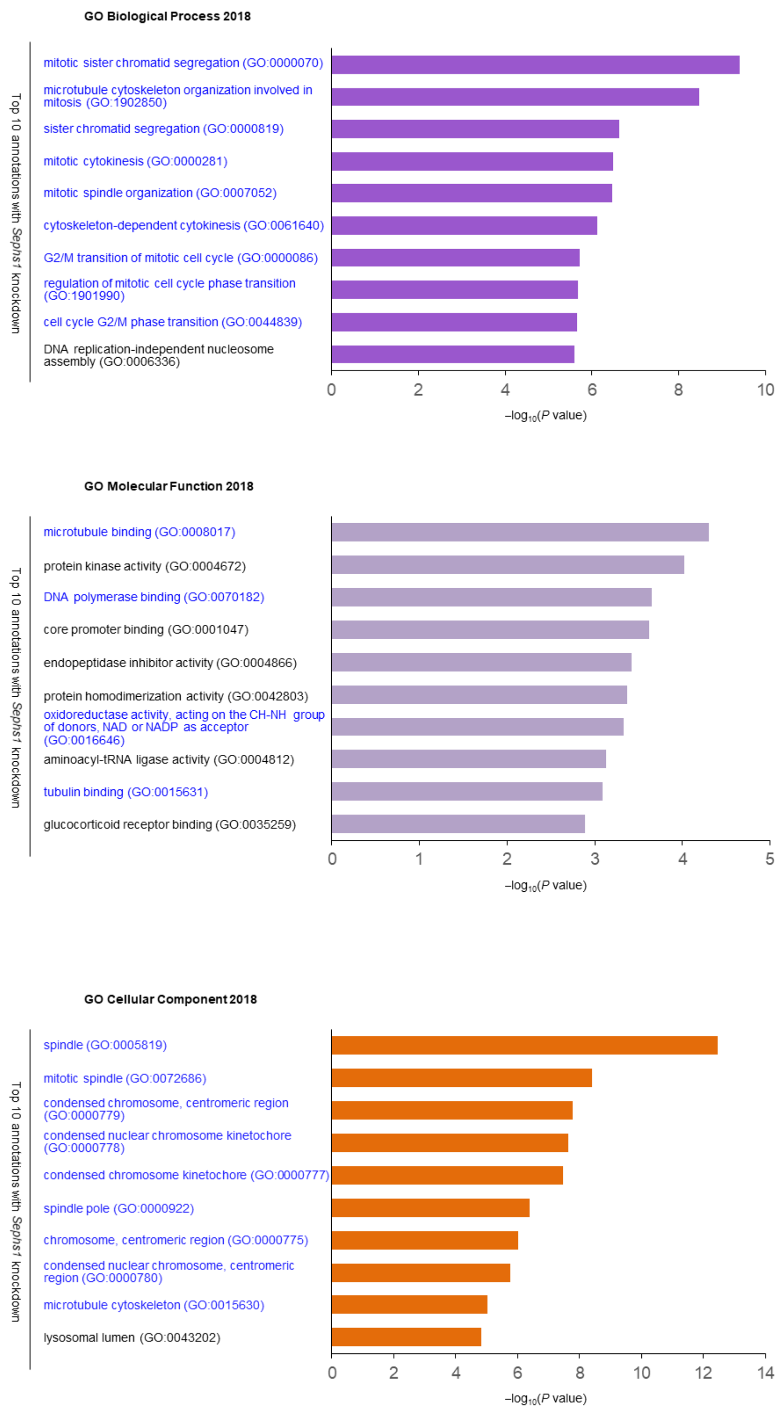

Supplementary Fig. 3 Transcriptome analysis reveals that loss of SEPHS1 activates ontologies related to cell cycle regulation, chromosome segregation, and oxidoreductase activity in chondrocytes. Top 10 annotations of differentially expressed genes following knockdown of *Sephs1* in chondrocytes from Enrichr Ontologies analysis, based on three Gene Ontology (GO) databases; the corresponding $-\log_{10}$(P values) are presented as graphs. P values are from Fisher's exact test.

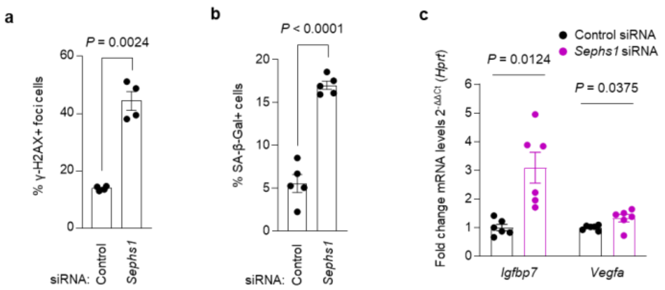

Supplementary Fig. 4 Downregulation of SEPHS1 in chondrocytes leads to cellular senescence and upregulation of SASPs expression. **a**, **b** Quantification of **a** immunofluorescence positivity of γ-H2AX (n = 4) or **b** SA-β-Gal positivity (n = 5) in primary cultured chondrocytes transfected with negative control siRNA or siRNA targeting *Sephs1*. **c** Relative mRNA expression of non-MMP families of SASP factors in chondrocytes transfected with negative control siRNA or siRNA targeting *Sephs1* (n = 6). **a**–**c** Data represent means ± s.e.m. P values are from two-tailed t test (**a**–**c**).

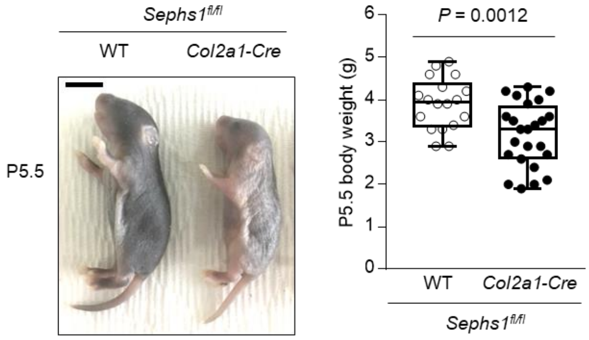

Supplementary Fig. 5 Loss of SEPHS1 during skeletal development causes growth retardation in mice. Representative photograph and quantification of body weight on postnatal day 5.5 (P5.5) in *Sephs1*$^{fl/fl}$ (*n* = 18) and *Sephs1*$^{fl/fl}$*; Col2a1-Cre* mice (*n* = 24). Scale bar: 1 cm. Data represent means ± s.e.m. *P* values are from two-tailed *t* test. The box and whiskers plot shows median values (center line) and the 25th (bottom line) and 75th percentiles (top line) with whiskers indicating the range.

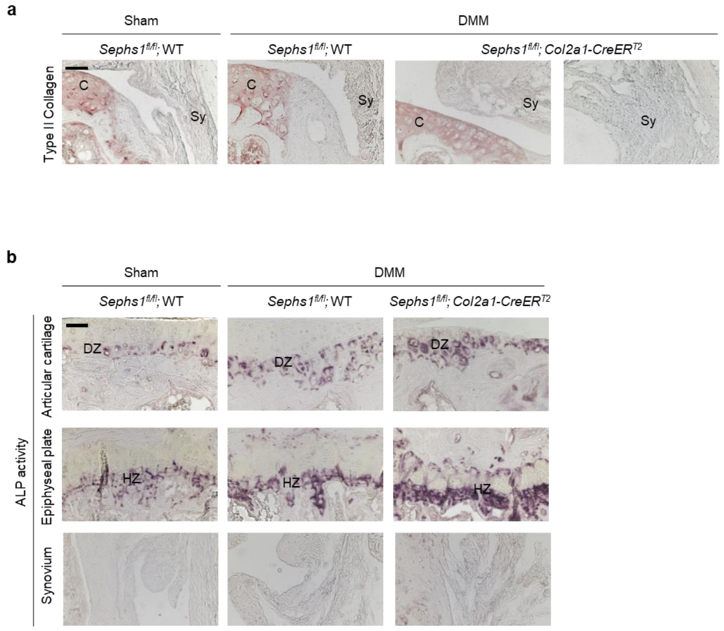

Supplementary Fig. 6 Synovial ectopic calcification is not observed in WT and SEPHS1-deficient mice. **a** Type II collagen was detected by immunohistochemistry in knee joint sections of sham- or DMM-operated WT and *Sephs1*-iCKO mice. **b** ALP activity was examined using NBT/BCIP substrates in knee joint sections of WT and *Sephs1*-iCKO mice. ALP activity was detected in the deep zone of the articular cartilage and in the hypertrophic zone of the epiphyseal plate, but not in the synovium. Scale bars: **a**, **b** 50 μm. Abbreviations: C, cartilage; Sy, synovium; DZ, deep zone; HZ, hypertrophic zone.

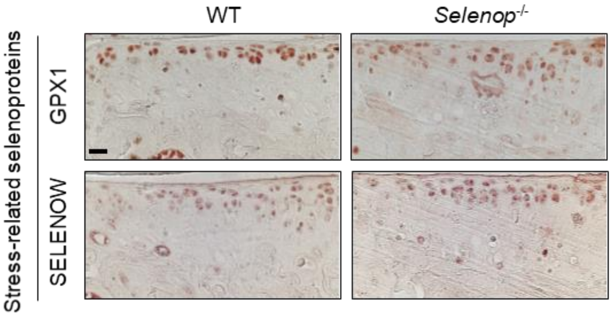

Supplementary Fig. 7 Immunohistochemical staining of stress-related selenoproteins in cartilage sections of *Selenop* KO mice. GPX1 and SELENOW were detected by immunohistochemistry in cartilage sections from the knee joints of 12-week-old WT and *Selenop* KO mice. Because SELENOP is the major selenium transporter protein that delivers selenium throughout the body, we used *Selenop* KO mice to mimic a low circulating selenium condition. Interestingly, the expression levels of GPX1 and SELENOW were not significantly changed in the cartilage of *Selenop* KO mice compared to those of WT mice. Scale bar: 25 μm.

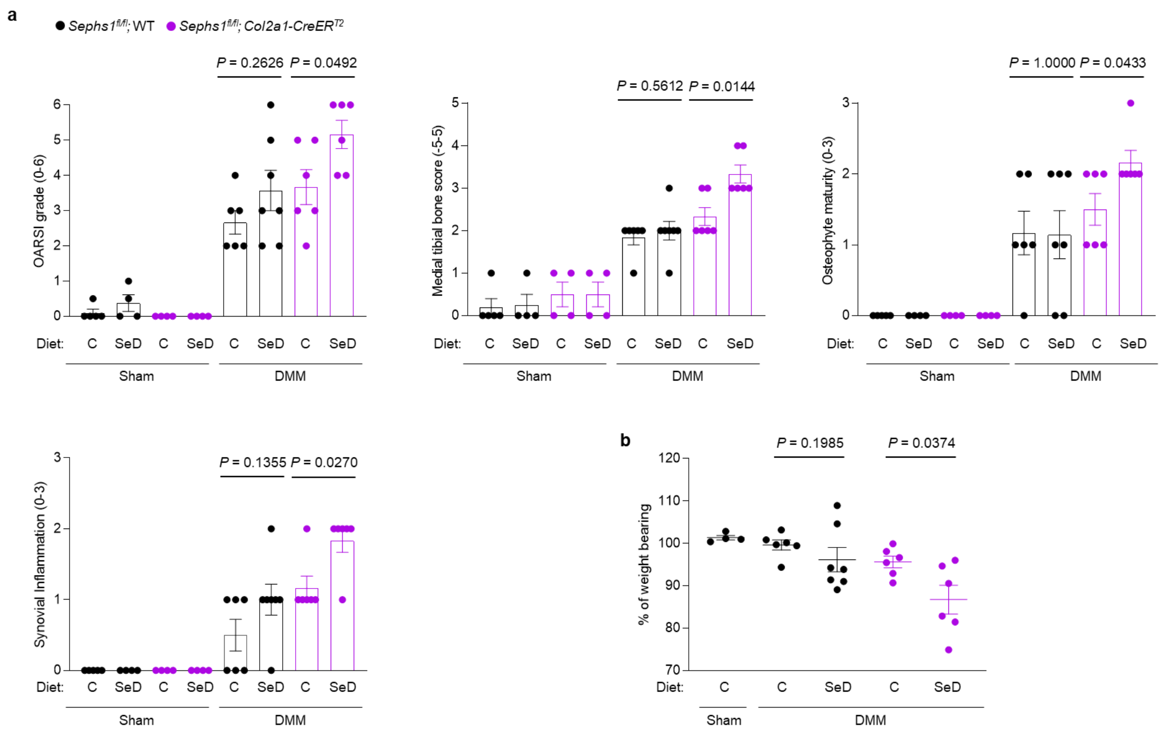

Supplementary Fig. 8 A selenium-deficient diet exacerbates the progression of OA in *Sephs1*-iCKO mice, but has no effect on sham surgery in WT or *Sephs1*-iCKO mice. **a** Cartilage destruction, subchondral bone sclerosis, osteophyte formation, and synovial inflammation determined by safranin O/hematoxylin staining and scored (n = 5 for sham-operated WT mice fed the control diet; n = 4 for sham-operated WT mice fed the selenium-deficient diet; n = 4 for sham-operated *Sephs1*-iCKO mice fed the control diet; n = 4 for sham-operated *Sephs1*-iCKO mice fed the selenium-deficient diet; n = 6 for DMM-operated WT fed the control diet; n = 7 for DMM-operated WT mice fed the selenium-deficient diet; n = 6 for DMM-operated *Sephs1*-iCKO mice fed the control diet; n = 6 for DMM-operated *Sephs1*-iCKO mice fed the selenium-deficient diet). **b** Percentage of weight placed on the sham- or DMM-operated limb versus the contralateral limb over 15 min analyzed by a dynamic weight bearing test (n = 4, 6, 7, 6, 6 respectively). **a**, **b** Data represent means ± s.e.m. P values are from S–R–H test followed by Mann–Whitney U test (**a**, **b**).

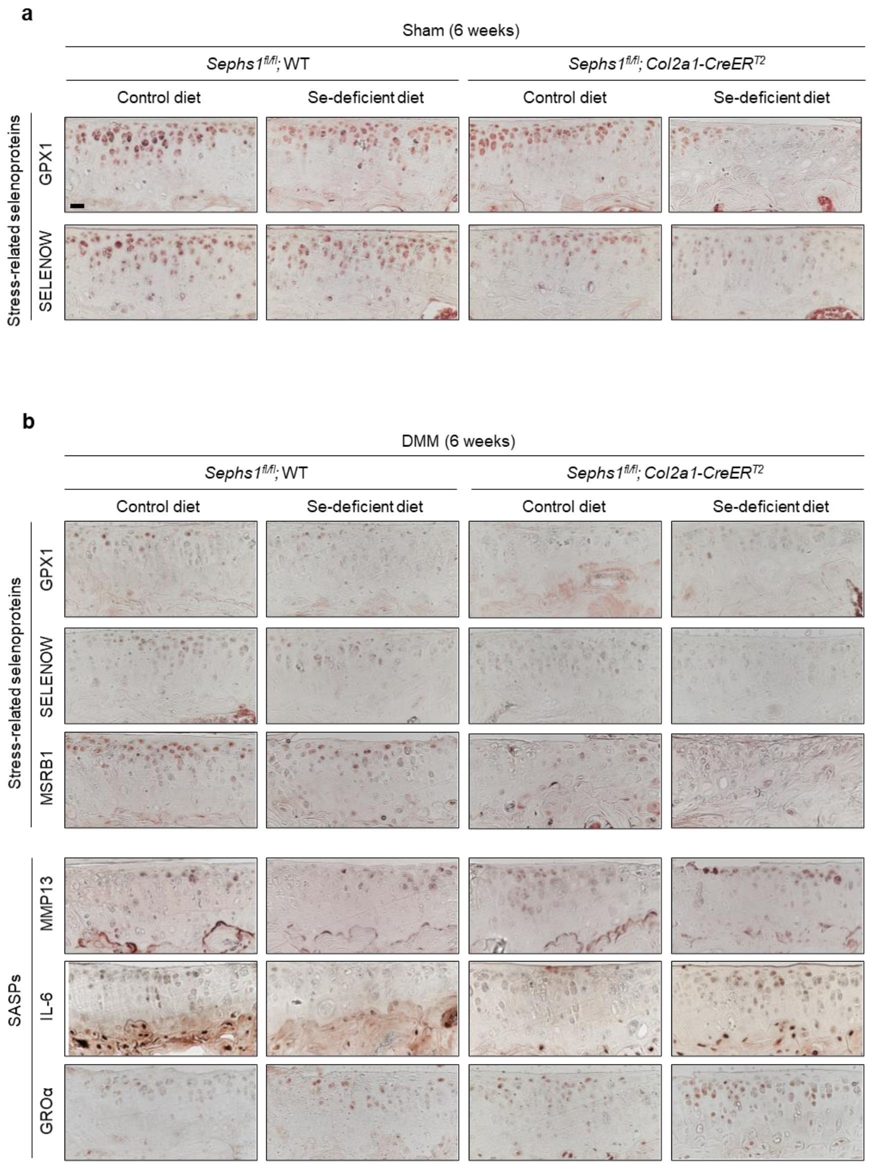

Supplementary Fig. 9 Immunohistochemical staining of stress-related selenoproteins and SASPs in cartilage sections of WT and SEPHS1-deficient mice fed a normal or selenium-deficient diet. **a** Stress-related selenoproteins (GPX1 and SELENOW) were detected by immunohistochemistry in cartilage sections from the knee joints of sham-operated WT and *Sephs1*-iCKO mice fed the indicated diets. **b** Stress-related selenoproteins (GPX1, SELENOW, and MSRB1) and SASPs (MMP13, IL-6, and GROα) were detected by immunohistochemistry in cartilage sections from the knee joints of DMM-operated WT and *Sephs1*-iCKO mice fed the indicated diets. Scale bar: **a** 25 μm.

Fig. 2b

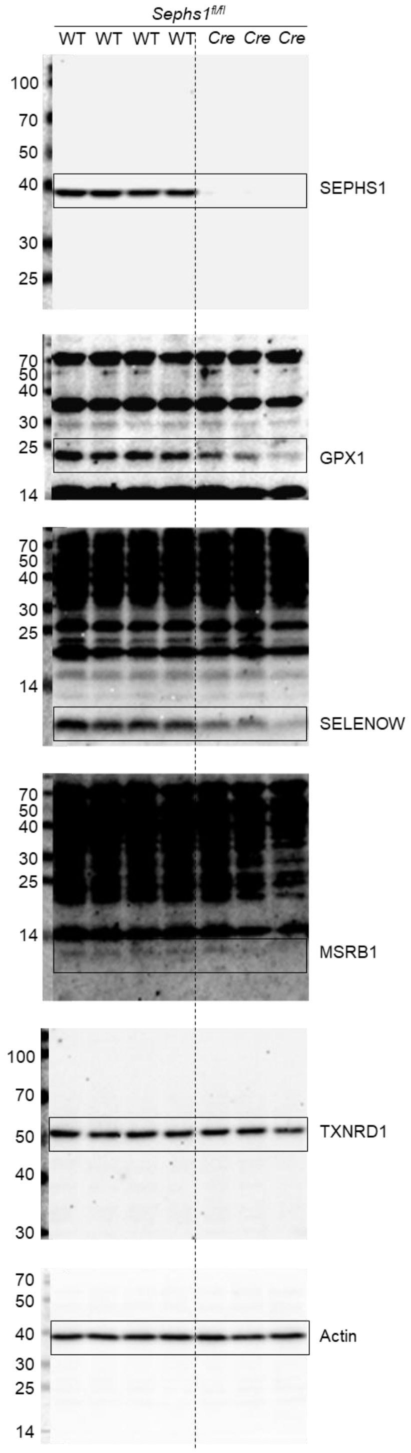

Supplementary Fig. 10 Unprocessed immunoblot images.

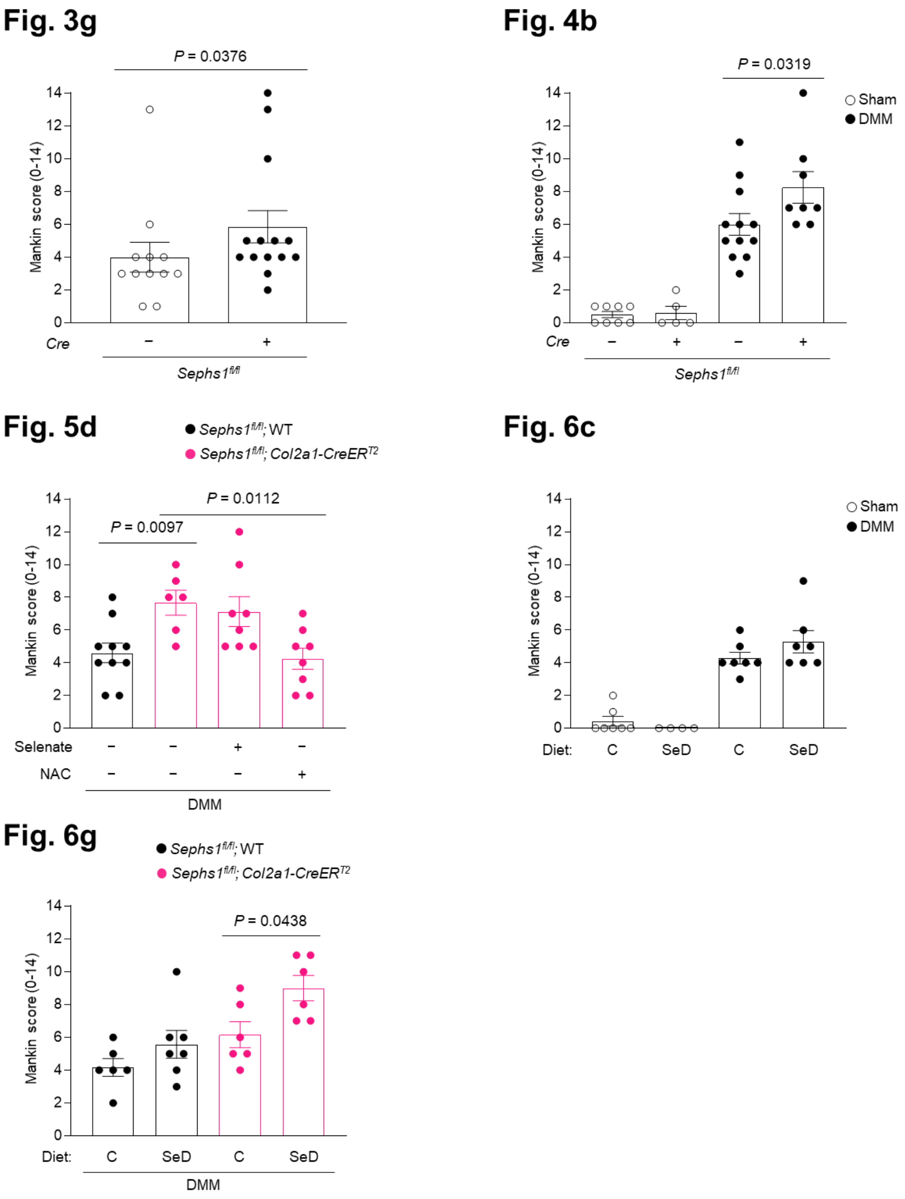

Supplementary Fig. 11 Mankin scores of *in vivo* data. Data represent means ± s.e.m. *P* values are from Mann–Whitney *U* test, Kruskal–Wallis test followed by Mann–Whitney *U* test, or S–R–H test followed by Mann–Whitney *U* test.

Fig. 3g

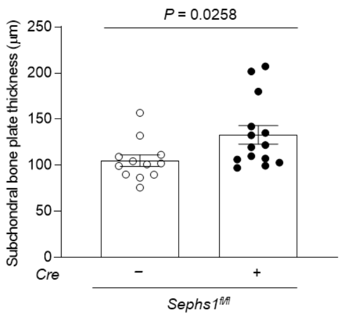

Fig. 4b

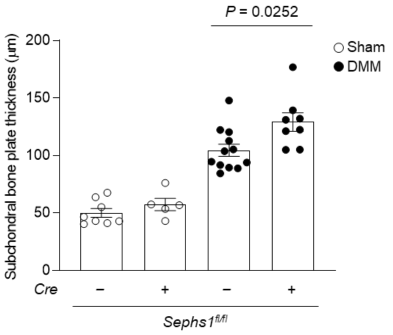

Fig. 5d

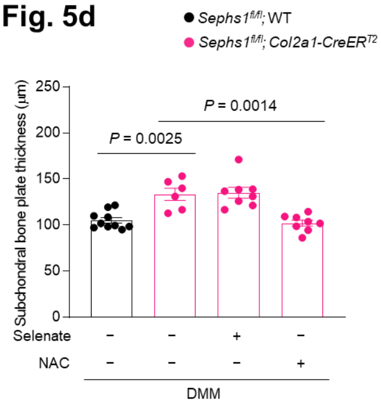

Fig. 6c

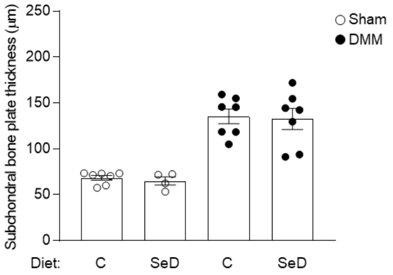

Fig. 6g

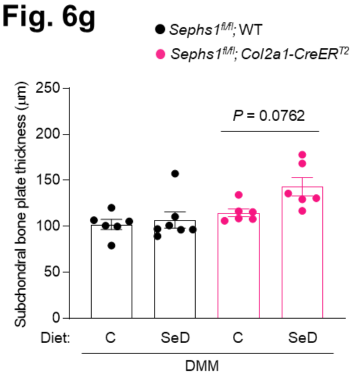

Supplementary Fig. 12 Subchondral bone plate thickness of the medial tibial plateau in the mouse model of post-traumatic OA. Data represent means ± s.e.m. *P* values are from *t* test or two-way ANOVA followed by Tukey's post-hoc test.

(n = 22)	Sex	Age (years)	Height (cm)	Weight (kg)	BMI (kg/m^2)
Range	22 Female	58–78	142.9–173.7	49.0–77.9	20.6–32.8
Average	-	68.4	152.8	63.7	27.3
SE	-	1.16	1.31	1.77	0.66

Supplementary Table 1 Descriptive characteristics of patients with OA. Abbreviation: BMI, body mass index.

Genes	Strand	Primer sequences
Cre	S	5′-GCGGTCTGGCAGTAAAAACTATC-3′
	AS	5′-GTGAAACAGCATTGCTGTCACTT-3′
*Sephs1*_floxed	S	5′-GAGATGCGTTTGTGTCCTCC-3′
	AS	5′-AGTGAGTGCCCGCCTTTA-3′
*Sephs1*_recombination efficiency	S	5′-CATCCTCCGTGATTCCCCTG-3′
	AS	5′-AACAGCTCCCAGAAACTGCT-3′

Supplementary Table 2 List of primers used for genotyping and validation of inducible knockout in *Sephs1* knockout mice. Abbreviations: S, sense strand; AS, antisense strand.

Genes	Strand	siRNA, miRNA, antimiR sequences	Species
Sephs1 #1	S	5′-GAGUGAUCCUGUUAUCCAAdTdT-3′	Mouse
	AS	5′-UUGGAUAACAGGAUCACUCdTdT-3′	
Sephs1 #3	S	5′-CGUAGUCAGAGGGUUGCAUdTdT-3′	Mouse
	AS	5′-AUGCAACCCUCUGACUACGdTdT-3′	

Supplementary Table 3 List of siRNAs. Abbreviations: S, sense strand; AS, antisense strand.

Genes	Strand	Primer sequences	Species
Hprt	S	5′-AGTCCCAGCGTCGTGATTAG-3′	Mouse
	AS	5′-GTATCCAACACTTCGAGAGGTC-3′	
Igfbp7	S	5′-CTCGCATCCAGCCACCTTAT-3′	Mouse
	AS	5′-ATGGAAGGACCTTGCTCGC-3′	
Mmp3	S	5′-TTGATGGGCCTGGAACAGTC-3′	Mouse
	AS	5′-AGTCCTGAGAGATTTGCGCC-3′	
Mmp10	S	5′-GTTCCTGTGTTGTCTGTCTCTC-3′	Mouse
	AS	5′-TGTTGCTCTTCAGTATGTGTGT-3′	
Mmp13	S	5′-TTCTTTGGCTTAGAGGTGACTG-3′	Mouse
	AS	5′-ACTGCTTGTCCAGGTTTCATC-3′	
Mmp14	S	5′-GACATCTTCTTGGTGGCTGTG-3′	Mouse
	AS	5′-CCCAGTGCTTATCTCCTTTGA-3′	
Sephs1	S	5′-GAGAGTCCTTTAACCCGGAG-3′	Mouse
	AS	5′-GCAAGTATCCATCCCAATGC-3′	
Vegfa	S	5′-ACTTTCTGCTCTCTTGGGTG-3′	Mouse
	AS	5′-CTGGCTTTGTTCTGTCTTTCTT-3′	

Supplementary Table 4 List of primers used in PCR. Abbreviations: S, sense strand; AS, antisense strand.

GO_Cellular senescence						
ABL1	*CDKN2A*	*ING2*	*MIR146A*	*NEK6*	*PRMT6*	*TERF2*
AKT3	*CDKN2B*	*KAT6A*	*MIR17*	*NSMCE2*	*RBL1*	*TERT*
ARG2	*CGAS*	*KIR2DL4*	*MIR188*	*NUAK1*	*RSL1D1*	*TP53*
ARNTL	*EEF1E1*	*KRAS*	*MIR20B*	*OPA1*	*SIRT1*	*TWIST1*
BCL2L12	*FBXO5*	*MAGEA2*	*MIR217*	*PAWR*	*SLC30A10*	*ULK3*
BCL6	*H2AFX*	*MAGEA2B*	*MIR22*	*PLA2R1*	*SMC5*	*VASH1*
BMPR1A	*HLA-G*	*MAP2K1*	*MIR34A*	*PLK2*	*SMC6*	*WNT16*
C2orf40	*HMGA1*	*MAP3K3*	*MIR543*	*PML*	*SRF*	*YPEL3*
CALR	*HMGA2*	*MAPK14*	*MIR590*	*PNPT1*	*TBX2*	*ZKSCAN3*
CDK6	*HRAS*	*MAPKAPK5*	*NAMPT*	*PRKCD*	*TBX3*	*ZMPSTE24*
CDKN1A	*ID2*	*MIR10A*	*NEK4*	*PRKDC*	*TERC*	*ZNF277*

Supplementary Table 5 Gene list of the ‘GO_Cellular senescence’ gene set.

Oxidative stress induced senescence (Reactome)						
AGO1	*EZH2*	*HIST1H2BD*	*HIST1H3I*	*HIST2H3C*	*MAPK14*	*RING1*
AGO3	*FOS*	*HIST1H2BE*	*HIST1H3J*	*HIST2H3D*	*MAPK3*	*RNF2*
AGO4	*H2AFB1*	*HIST1H2BF*	*HIST1H4A*	*HIST2H4A*	*MAPK8*	*RPS27A*
BMI1	*H2AFJ*	*HIST1H2BG*	*HIST1H4B*	*HIST2H4B*	*MAPK9*	*SCMH1*
CBX2	*H2AFV*	*HIST1H2BH*	*HIST1H4C*	*HIST3H2BB*	*MAPKAPK2*	*SUZ12*
CBX4	*H2AFX*	*HIST1H2BI*	*HIST1H4D*	*HIST4H4*	*MAPKAPK3*	*TFDP1*
CBX6	*H2AFZ*	*HIST1H2BJ*	*HIST1H4E*	*IFNB1*	*MAPKAPK5*	*TFDP2*
CBX8	*H2BFS*	*HIST1H2BK*	*HIST1H4F*	*JUN*	*MDM2*	*TNIK*
CDK4	*H3F3A*	*HIST1H2BL*	*HIST1H4H*	*KDM6B*	*MDM4*	*TNRC6A*
CDK6	*H3F3B*	*HIST1H2BM*	*HIST1H4I*	*MAP2K3*	*MINK1*	*TNRC6B*
CDKN2A	*HIST1H2AB*	*HIST1H2BN*	*HIST1H4J*	*MAP2K4*	*MIR24-1*	*TNRC6C*
CDKN2B	*HIST1H2AC*	*HIST1H2BO*	*HIST1H4K*	*MAP2K6*	*MIR24-2*	*TP53*
CDKN2C	*HIST1H2AD*	*HIST1H3A*	*HIST1H4L*	*MAP2K7*	*MOV10*	*TXN*
CDKN2D	*HIST1H2AE*	*HIST1H3D*	*HIST2H2AA3*	*MAP3K5*	*PHC1*	*UBA52*
E2F1	*HIST1H2AJ*	*HIST1H3E*	*HIST2H2AA4*	*MAP4K4*	*PHC2*	*UBB*
E2F2	*HIST1H2BA*	*HIST1H3F*	*HIST2H2AC*	*MAPK1*	*PHC3*	*UBC*
E2F3	*HIST1H2BB*	*HIST1H3G*	*HIST2H2BE*	*MAPK10*	*RBBP4*	
EED	*HIST1H2BC*	*HIST1H3H*	*HIST2H3A*	*MAPK11*	*RBBP7*	

Supplementary Table 6 Gene list of the ‘Reactome_Oxidative stress induced senescence’ gene set.

Upregulated genes in OA						
ABHD2	*CCND1*	*ENOX1*	*ID2*	*MYBL1*	*RAP2B*	*STXBP6*
ABI3BP	*CCNE2*	*EPDR1*	*IER3*	*MYC*	*RARB*	*SYN1*
ABRACL	*CCNYL1*	*EPHA3*	*IGF2BP2*	*MYO1B*	*RARRES1*	*SYNDIG1*
AC005082.12	*CD163L1*	*ESPL1*	*IGFBP3*	*NAV3*	*RASGEF1B*	*SYT11*
AC007362.1	*CD300C*	*ESRRA*	*IGFBP4*	*NBL1*	*RCAN1*	*SYTL2*
AC009299.3	*CD55*	*ETV4*	*IGFBP7*	*NCAPG*	*RGCC*	*TACSTD2*
AC074093.1	*CD58*	*EVA1A*	*IGSF3*	*NCF2*	*RHBDL2*	*TAGLN2*
AC093850.2	*CD68*	*EVI2A*	*IL11*	*NEDD4L*	*RHPN2*	*TAGLN2P1*
AC144831.1	*CDC20*	*EVI2B*	*IL13RA2*	*NEDD9*	*RIPK3*	*TBC1D7*
ADAM12	*CDC6*	*EVL*	*IL8*	*NETO2*	*RIPK4*	*TBC1D9*
ADAM9	*CDH10*	*EYA4*	*INHBA*	*NFIL3*	*RND1*	*TBX3*
ADAMTS1	*CDH19*	*EZR*	*INHBB*	*NFKBIZ*	*RND3*	*TBX5*
ADAMTS12	*CDH2*	*F11R*	*IQGAP3*	*NGF*	*RNF128*	*TBX5-AS1*
ADAMTS14	*CDK1*	*F13A1*	*IRX2*	*NKX2-5*	*RNF152*	*TCAIM*
ADAMTS5	*CDK6*	*F2R*	*IRX5*	*NOS2*	*RNF180*	*TCEAL7*
ADAMTS6	*CDKN1A*	*F3*	*ISG15*	*NOVA1*	*RNPEPL1*	*TCIRG1*
ADTRP	*CDKN2B*	*FAM111B*	*ISM2*	*NPR3*	*ROR1*	*TENM2*
AFAP1L1	*CDKN3*	*FAM126A*	*ITGA3*	*NPTX2*	*RP11-143E21.7*	*TENM3*
AGXT2L1	*CELF2*	*FAM132B*	*ITGA4*	*NRIP3*	*RP11-150O12.1*	*TES*
AHR	*CENPE*	*FAM134B*	*ITGA9*	*NRP2*	*RP11-160A10.2*	*TFPI*
AKR1C1	*CENPF*	*FAM167A*	*ITGAX*	*NT5E*	*RP11-18F14.2*	*TFPI2*
AKR1C2	*CENPH*	*FAM173B*	*ITGB4*	*NTF3*	*RP11-215A21.2*	*TGFBI*
AKR1C3	*CENPK*	*FAM180A*	*ITGB5*	*NTRK2*	*RP11-267A15.1*	*TGFBR1*
AL139147.1	*CENPP*	*FAM60A*	*ITPR1*	*NUDT11*	*RP11-282K24.1*	*THY1*
ALDH3B1	*CEP55*	*FAM89B*	*KAZN*	*NUSAP1*	*RP11-316P21.1*	*TIMP3*
ALS2CL	*CGRRF1*	*FANCI*	*KCNA1*	*NXPE2*	*RP11-350G8.3*	*TLR6*
ALS2CR11	*CHML*	*FAP*	*KCNE4*	*OCIAD2*	*RP11-363J20.2*	*TM4SF1*
AMPH	*CHRD*	*FAT3*	*KCNG2*	*ODF3B*	*RP11-383H13.1*	*TMEM100*
ANGPTL1	*CHST13*	*FBLN2*	*KCNN4*	*OGN*	*RP11-456H18.2*	*TMEM119*
ANK3	*CHST15*	*FBXO16*	*KCNS3*	*OLFML2B*	*RP11-536I6.2*	*TMEM126A*
ANKRD44	*CITED4*	*FGF9*	*KIAA1217*	*OMD*	*RP11-556K13.1*	*TMEM150C*
ANKRD9	*CKAP2*	*FHL2*	*KIAA1244*	*OPN3*	*RP11-73E17.2*	*TMEM154*
ANLN	*CKAP2L*	*FN1*	*KIF11*	*ORMDL2*	*RP11-841C19.6*	*TMEM200A*
ANO5	*CKB*	*FNDC1*	*KIF18A*	*OSBPL3*	*RP2*	*TMEM59L*
ANPEP	*CKS2*	*FNIP2*	*KIF20A*	*OSTC*	*RPL22L1*	*TMOD1*
ANXA1	*CLCF1*	*FOSL1*	*KIF23*	*OTUD1*	*RPL29P33*	*TMOD3*
ANXA8	*CLDN1*	*FOXF1*	*KIF5C*	*P4HA3*	*RPSAP4*	*TMSB4X*
ANXA8L1	*CLDN7*	*FOXM1*	*KIFC1*	*PAMR1*	*RPSAP58*	*TMSB4XP1*
ANXA8L2	*CLIC3*	*FRMD6*	*KL*	*PAPPA*	*RRM2*	*TMSB4XP2*
AOC2	*CLIC6*	*FRRS1*	*KLF6*	*PARP8*	*RTN2*	*TMSB4XP6*
AOC3	*CNIH*	*FSTL1*	*KLF7*	*PAWR*	*S100A2*	*TMSB4XP8*
AP000330.8	*CNKSR2*	*FSTL3*	*KLF9*	*PAX1*	*S100A3*	*TNFAIP6*
APCDD1L	*CNTN1*	*FTCD*	*KLHL35*	*PBK*	*S100A4*	*TNFRSF11A*
APOBEC3C	*COL13A1*	*FZD1*	*KLHL5*	*PCDH1*	*S100A6*	*TNFRSF11B*

AQP1	*COL15A1*	*FZD10*	*KLRD1*	*PCDH10*	*S1PR3*	*TNFRSF12A*
ARG2	*COL18A1*	*FZD3*	*KPNA2*	*PCDH18*	*SAMD9*	*TNFSF10*
ARHGAP11A	*COL1A1*	*GADD45B*	*LACC1*	*PCSK5*	*SCD5*	*TNFSF11*
ARHGAP24	*COL22A1*	*GALNT12*	*LAMA5*	*PDE10A*	*SCO2*	*TNNI2*
ARHGAP28	*COL24A1*	*GALNT13*	*LAMB3*	*PDGFA*	*SCXA*	*TOM1L1*
ARHGAP44	*COL25A1*	*GALNT16*	*LAMC2*	*PDGFC*	*SCXB*	*TOP2A*
ARHGAP9	*COL5A3*	*GALNT7*	*LANCL3*	*PDLIM7*	*SDK1*	*TPX2*
ARID5B	*COL6A3*	*GAS1*	*LEFTY2*	*PERP*	*SEMA3C*	*TRAK1*
ARL4A	*COL7A1*	*GAS2L3*	*LEPREL1*	*PGM2L1*	*SEMA4D*	*TREM1*
ARL4C	*COL8A1*	*GDF6*	*LHX9*	*PGR*	*SEMA5A*	*TRIM36*
ARNTL2	*COL8A2*	*GFRA2*	*LIF*	*PHLDA2*	*SERINC2*	*TRPM8*
ARSI	*CORO1C*	*GGH*	*LINC00152*	*PI4K2B*	*SERPINB8*	*TSC22D3*
ASAP2	*CPEB2*	*GINS2*	*LINC00467*	*PIM1*	*SERPINE1*	*TSPAN12*
ASPM	*CRLF1*	*GINS4*	*LINC00517*	*PITPNM3*	*SERPINE2*	*TSPAN2*
ASPN	*CRNDE*	*GIPR*	*LINC00545*	*PLAT*	*SERPINF1*	*TSTD1*
ATAD5	*CRTAC1*	*GJA1*	*LINC00607*	*PLAUR*	*SERTAD1*	*TTC9*
ATP6AP2	*CSDC2*	*GJB2*	*LMNB1*	*PLD1*	*SGK1*	*TTK*
ATRNL1	*CSRNP1*	*GJC1*	*LMO2*	*PLEKHF2*	*SGMS2*	*TUBA1A*
B3GNT2	*CTC-298B17.1*	*GLIS1*	*LMX1B*	*PLEKHG1*	*SGOL2*	*TYMP*
B3GNT5	*CTD-2319I12.1*	*GLIS3*	*LOXL1*	*PLK4*	*SH3KBP1*	*TYMS*
BAALC	*CXCR7*	*GLP2R*	*LRRC8B*	*PLS3*	*SHC4*	*TYRO3*
BACH1	*CXorf57*	*GLRB*	*LRRC8C*	*PLXNA2*	*SHCBP1*	*UAP1*
BAG2	*CYB5R4*	*GLRX*	*LRRC8E*	*PLXNA4*	*SIK1*	*UBAC2*
BARX2	*CYFIP2*	*GLRX3*	*LRRFIP1*	*PMAIP1*	*SIPA1L2*	*UBE2D1*
BCAS4	*CYP27B1*	*GLYATL2*	*LTBP4*	*PNMA2*	*SIX1*	*UBE2T*
BDNF	*DBNDD1*	*GMNN*	*LUM*	*PNP*	*SKAP2*	*UGP2*
BIRC5	*DDHD1*	*GNAI1*	*LY6D*	*PODXL*	*SLC16A10*	*UHRF1*
BIRC7	*DENND3*	*GNG11*	*LY96*	*PODXL2*	*SLC24A3*	*ULBP1*
BMP2K	*DEPDC1*	*GNPNAT1*	*LYPLA1*	*POPDC3*	*SLC2A12*	*ULBP2*
BMPR1B	*DGKI*	*GPC4*	*MAD2L1*	*POSTN*	*SLC2A5*	*UPK1B*
BPGM	*DIAPH3*	*GPM6B*	*MAGED4B*	*PPP1R14C*	*SLC30A1*	*UROC1*
BRCA2	*DIRAS1*	*GPR176*	*MAMDC2*	*PPP1R36*	*SLC31A2*	*VANGL1*
BTBD16	*DIXDC1*	*GPR183*	*MAP1A*	*PPTC7*	*SLC35E4*	*VCAN*
BUB1	*DKK3*	*GPR56*	*MAP1B*	*PQLC3*	*SLC38A5*	*VEGFC*
BUB1B	*DLGAP5*	*GPR64*	*MAP7*	*PRC1*	*SLC39A14*	*VEPH1*
BVES	*DLX4*	*GRIA2*	*MAPKAPK3*	*PRDM1*	*SLC41A2*	*VPS13A*
BZW2	*DNAJC12*	*GRIP1*	*MARC2*	*PREX2*	*SLC44A5*	*VSIG2*
C10orf105	*DNAJC22*	*GSKIP*	*MARCKS*	*PRKAR2B*	*SLC4A7*	*VSNL1*
C11orf82	*DNER*	*GTDC1*	*MARS2*	*PROCR*	*SLC6A6*	*VWC2*
C12orf5	*DOK6*	*H2AFZ*	*MBP*	*PRSS23*	*SLC7A2*	*WDR69*
C1GALT1	*DOPEY2*	*HBA2*	*MELK*	*PSAT1*	*SLC7A5*	*WISP1*
C1GALT1C1	*DPP4*	*HBB*	*MEOX2*	*PTGER1*	*SLFN11*	*WNT16*
C1orf114	*DSG2*	*HEBP2*	*MET*	*PTGER2*	*SLITRK6*	*WNT5A*
C1QTNF1	*DTL*	*HEG1*	*MEX3D*	*PTGER4*	*SLMO2*	*WNT5B*
C1QTNF2	*DUSP4*	*HES6*	*MFSD6*	*PTGES*	*SMIM5*	*WNT7B*

C1QTNF3	*DUSP5*	*HEY2*	*MICAL2*	*PTGFR*	*SNHG5*	*WNT9A*
C1QTNF7	*DUSP6*	*HHIPL1*	*MINOS1*	*PTGS2*	*SNTB1*	*YRDC*
C3orf14	*DUSP8*	*HJURP*	*MIR31HG*	*PTPRD*	*SNX10*	*YY2*
C3orf52	*DYNLT3*	*HMCN1*	*MKI67*	*PTPRK*	*SNX7*	*ZBTB21*
C4orf48	*DYSF*	*HMGA1*	*MLF1IP*	*PTTG1*	*SOD2*	*ZDHHC2*
C5orf38	*E2F1*	*HMGA2*	*MMP28*	*PTX3*	*SORBS2*	*ZIC1*
C6orf132	*EAF2*	*HMGB3P10*	*MOB3B*	*PXDN*	*SORT1*	*ZIC4*
CA12	*EBF3*	*HMMR*	*MOXD1*	*QPCT*	*SOWAHC*	*ZNF277*
CACHD1	*ECT2*	*HOMER2*	*MPP7*	*R3HDML*	*SOX11*	*ZNF365*
CACNA1A	*EDEM2*	*HOXB2*	*MSC*	*RAB23*	*SPAG1*	*ZNF367*
CAPS	*EDNRA*	*HOXB-AS1*	*MSX2*	*RAB31*	*SPECC1*	*ZNF503-AS1*
CASC5	*EEF1A1P12*	*HPSE2*	*MT1A*	*RAB32*	*SPINT1*	*ZNF544*
CASP4	*EEF1A1P33*	*HS3ST2*	*MT1F*	*RAB38*	*SPRY1*	*ZNF883*
CBR3	*EEF1E1*	*HSD17B11*	*MT1G*	*RABIF*	*SPSB4*	*ZWILCH*
CCDC109B	*EFHD2*	*HSD3B7*	*MT1L*	*RAC2*	*SQRDL*	*ZWINT*
CCDC112	*EGR2*	*HSPB8*	*MT1M*	*RACGAP1*	*ST3GAL1*	
CCL20	*EHD4*	*HTRA1*	*MTHFD1L*	*RAD51*	*ST6GAL2*	
CCNB1	*ELMO1*	*HUNK*	*MTSS1*	*RAD51AP2*	*ST6GALNAC5*	
CCNB2	*EMP1*	*ICA1*	*MUC12*	*RAI14*	*STX1A*	

Supplementary Table 7 Gene list of the ‘Upregulated genes in OA’ gene set[30].

Fig.	Measure	Groups compared (control vs. experimental)	Mean difference	Pooled SD	Cohen's *d* (magnitude)	95% CI of difference between means
1b	OARSI grade	Undamaged vs. Damaged	2.318	0.798	2.904 (huge)	2.526 to 3.281
1e	OARSI grade	2-month-old vs. 24-month-old	2.250	0.53	4.243 (huge)	1.206 to 7.279
1f	OARSI grade	Sham vs. DMM	3.389	0.894	3.791 (huge)	2.156 to 5.426
3g	OARSI grade	WT vs. iCKO	1.030	1.667	0.618 (medium)	0.283 to 0.952
	Medial tibial bone score	WT vs. iCKO	1.595	1.252	1.275 (very large)	0.891 to 1.658
	Osteophyte maturity	WT vs. iCKO	0.798	0.744	1.073 (large)	0.707 to 1.438
	Synovial inflammation	WT vs. iCKO	0.452	0.613	0.738 (medium)	0.397 to 1.079
4b	OARSI grade	DMM WT vs. DMM iCKO	1.708	1.197	1.427 (very large)	0.882 to 1.972
	Medial tibial bone score	DMM WT vs. DMM iCKO	1.250	1.007	1.241 (very large)	0.722 to 1.760
	Osteophyte maturity	DMM WT vs. DMM iCKO	0.792	0.728	1.087 (large)	0.588 to 1.587
	Synovial inflammation	DMM WT vs. DMM iCKO	0.792	0.728	1.087 (large)	0.588 to 1.587
5d	OARSI grade	WT vs. iCKO	1.533	0.799	1.920 (very large)	1.101 to 2.739
	Medial tibial bone score	WT vs. iCKO	1.767	0.935	1.890 (very large)	1.079 to 2.701
	Osteophyte maturity	WT vs. iCKO	1.267	0.834	1.519 (very large)	0.792 to 2.246
	Synovial inflammation	WT vs. iCKO	0.800	0.507	1.578 (very large)	0.839 to 2.316
	OARSI grade	iCKO vs. iCKO+NAC	-1.333	0.882	-1.512 (very large)	-2.325 to -0.698
	Medial tibial bone score	iCKO vs. iCKO+NAC	-1.292	1.049	-1.231 (very large)	-1.985 to -0.478
	Osteophyte maturity	iCKO vs. iCKO+NAC	-1.417	0.833	-1.700 (very large)	-2.560 to -0.840
	Synovial inflammation	iCKO vs. iCKO+NAC	-1.000	0.540	-1.852 (very large)	-2.754 to 0.949
6g	OARSI grade	DMM iCKO C vs. DMM iCKO SeD	1.500	1.103	1.360 (very large)	0.446 to 2.274
	Medial tibial bone score	DMM iCKO C vs. DMM iCKO SeD	1.000	0.516	1.936 (very large)	0.846 to 3.027
	Osteophyte maturity	DMM iCKO C vs. DMM iCKO SeD	0.667	0.483	1.380 (very large)	0.461 to 2.300
	Synovial inflammation	DMM iCKO C vs. DMM iCKO SeD	0.667	0.408	1.633 (very large)	0.643 to 2.623

Supplementary Table 8 Cohen's *d* effect size measurements of statistically significant changes observed from *in vivo* data scored based on ordinal scoring systems. Abbreviation: CI, confidence interval.

Supplementary Note 1 The detailed results of statistical analyses using *t* test and two-way ANOVA, referring to Fig. 2a, k, l, m, Fig. 5b, and Supplementary Fig. 4c.

The details of statistical analyses for Fig. 2a, k, l, m, Fig. 5b, and Supplementary Fig. 4c are summarized in this file.

Fig. 2a Relative mRNA expression level of *Sephs1* in primary cultured chondrocytes isolated from *Sephs1*$^{fl/fl}$ or *Sephs1*$^{fl/fl}$*; Col2a1-Cre* mice (n = 4).

Two independent sample *t* test (equal variance)					
	Shapiro-Wilk test		Levene's test	*P*-value	95% CI of diff of means
	Sephs1 $^{f/f}$	*Col2a1-cre; Sephs1* $^{f/f}$			
Sephs1 $^{fl/fl}$ vs. *Col2a1-Cre; Sephs1* $^{fl/fl}$	W = 0.8836	W = 0.9396	F-value = 5.5856	*P*-value < 0.0001	0.8305 to 1.1482
	P-value = 0.3541	*P*-value = 0.6521	*P*-value = 0.0560		

Fig. 2k Quantification of immunofluorescence positivity of γ-H2AX in primary cultured chondrocytes transfected with negative control siRNA or siRNA targeting *Sephs1* followed by NAC treatment at the indicated doses (n = 4).

Two-way ANOVA with siRNA and treatment<br>siRNA : treatment interaction as factors<br>F(1,18) = 58.1002 for siRNA, *P*-value < 0.0001, df = 1<br>F(2,18) = 34.0279 for treatment, *P*-value < 0.0001, df = 2<br>F(2,18) = 26.7860 for siRNA : treatment, *P*-value < 0.0001, df = 2

Dunnett's post-hoc test		
Groups	*P*-value	Estimates 95% CI of diff
NAC 0 : si*Sephs1* vs NAC 0 : siNC	<0.0001	16.07 to 27.77
NAC 0 : si*Sephs1* vs NAC 0.5 : si*Sephs1*	<0.0001	12.63 to 24.33
NAC 0 : si*Sephs1* vs NAC 0.5 : siNC	<0.0001	16.74 to 28.44
NAC 0 : si*Sephs1* vs NAC 1 : si*Sephs1*	<0.0001	15.69 to 27.39
NAC 0 : si*Sephs1* vs NAC 1 : siNC	<0.0001	17.63 to 29.33

Fig. 2l Quantification of SA-β-Gal positivity in primary cultured chondrocytes transfected with negative control siRNA or siRNA targeting *Sephs1* followed by NAC treatment at the indicated doses (n = 4).

Two-way ANOVA with siRNA and treatment
siRNA : treatment interaction as factors
F(1,18) = 69.7372 for siRNA, *P*-value < 0.0001, df = 1
F(2,18) = 16.6613 for treatment, *P*-value < 0.0001, df = 2
F(2,18) = 22.6534 for siRNA : treatment, *P*-value < 0.0001, df = 2

Dunnett's post-hoc test		
Groups	*P*-value	Estimates 95% CI of diff
NAC 0 : si*Sephs1* vs NAC 0 : siNC	<0.0001	8.444 to 14.88
NAC 0 : si*Sephs1* vs NAC 0.5 : si*Sephs1*	<0.0001	3.231 to 9.671
NAC 0 : si*Sephs1* vs NAC 0.5 : siNC	<0.0001	7.682 to 14.12
NAC 0 : si*Sephs1* vs NAC 1 : si*Sephs1*	<0.0001	6.963 to 13.40
NAC 0 : si*Sephs1* vs NAC 1 : siNC	<0.0001	7.714 to 14.15

Fig. 2m and Supplementary Fig. 4c Relative mRNA expression of SASP factors in chondrocytes transfected with negative control siRNA or siRNA targeting *Sephs1* (n = 6).

Two independent sample *t* test (equal variance)					
Mmp3	Shapiro-Wilk test		Levene's test	*P*-value	95% CI of diff of means
	siNC	si*Sephs1*			
siNC vs. si*Sephs1*	W = 0.9407	W = 0.9574	F-value = 0.6864	*P*-value = 0.0123	0.2773 to 1.7878
	P-value = 0.6652	*P*-value = 0.7997	*P*-value = 0.4267		

Two independent sample *t* test (equal variance)					
Mmp10	Shapiro-Wilk test		Levene's test	*P*-value	95% CI of diff of means
	siNC	si*Sephs1*			
siNC vs. si*Sephs1*	W = 0.9547	W = 0.9565	F-value = 0.0253	*P*-value = 0.0084	0.2744 to 1.4435
	P-value = 0.7785	*P*-value = 0.7924	*P*-value = 0.8769		

Two independent sample *t* test (equal variance)					
Mmp13	Shapiro-Wilk test		Levene's test	*P*-value	95% CI of diff of means
	siNC	si*Sephs1*			
siNC vs. si*Sephs1*	W = 0.9237	W = 0.9814	F-value = 3.9749	*P*-value = 0.0051	0.3996 to 1.7250
	P-value = 0.5325	*P*-value = 0.9584	*P*-value = 0.0742		

Welch's *t* test (unequal variance)					
Mmp14	Shapiro-Wilk test		Levene's test	*P*-value	95% CI of diff of means
	siNC	si*Sephs1*			
siNC vs. si*Sephs1*	W = 0.9475	W = 0.8348	F-value = 6.0763	*P*-value = 0.0249	0.2223 to 1.5520
	P-value = 0.7203	*P*-value = 0.1181	*P*-value = 0.0334		

Welch's *t* test (unequal variance)					
Igfbp7	Shapiro-Wilk test		Levene's test	*P*-value	95% CI of diff of means
	siNC	si*Sephs1*			
siNC vs. si*Sephs1*	W = 0.9607	W = 0.8915	F-value = 24.4072	*P*-value = 0.0124	0.8711 to 3.3142
	P-value = 0.8251	*P*-value = 0.3259	*P*-value = 0.0006		

Two independent sample *t* test (equal variance)					
Vegfa	Shapiro-Wilk test		Levene's test	*P*-value	95% CI of diff of means
	siNC	si*Sephs1*			
siNC vs. si*Sephs1*	W = 0.9149	W = 0.8507	F-value = 3.0702	*P*-value = 0.0375	0.0229 to 0.6291
	P-value = 0.4668	*P*-value = 0.1595	*P*-value = 0.1103		

Fig. 5b Body weight of 21-week-old DMM-operated mice after completion of the supplementation scheme ($n \geq 6$).

Two-way ANOVA with genotype and treatment
F(2,28) = 0.7594 for treatment, *P*-value = 0.4773, df = 2
F(1,28) = 2.1090 for genotype, *P*-value = 0.1576, df = 1

No post-hoc test was performed after two-way ANOVA analysis.
