# Supplementary material for: Selenophosphate synthetase 1 deficiency exacerbates osteoarthritis by dysregulating redox homeostasis
Source: Nat Commun. 2022 Feb 9;13:779. doi: 10.1038/s41467-022-28385-7 (PMC8828855; doi:10.1038/s41467-022-28385-7)
Supplement: Supplementary file 2 — Reporting Summary [file 41467_2022_28385_MOESM2_ESM.pdf]

nature portfolio

Corresponding author(s): Jin-Hong Kim

Last updated by author(s): Dec 2, 2021

Reporting Summary

Nature Portfolio wishes to improve the reproducibility of the work that we publish. This form provides structure for consistency and transparency in reporting. For further information on Nature Portfolio policies, see our Editorial Policies and the Editorial Policy Checklist.

Statistics

For all statistical analyses, confirm that the following items are present in the figure legend, table legend, main text, or Methods section.

n/a	Confirmed	
☐	☒	The exact sample size (n) for each experimental group/condition, given as a discrete number and unit of measurement
☐	☒	A statement on whether measurements were taken from distinct samples or whether the same sample was measured repeatedly
☐	☒	The statistical test(s) used AND whether they are one- or two-sided<br>*Only common tests should be described solely by name; describe more complex techniques in the Methods section.*
☒	☐	A description of all covariates tested
☐	☒	A description of any assumptions or corrections, such as tests of normality and adjustment for multiple comparisons
☐	☒	A full description of the statistical parameters including central tendency (e.g. means) or other basic estimates (e.g. regression coefficient) AND variation (e.g. standard deviation) or associated estimates of uncertainty (e.g. confidence intervals)
☐	☒	For null hypothesis testing, the test statistic (e.g. F, t, r) with confidence intervals, effect sizes, degrees of freedom and P value noted<br>*Give P values as exact values whenever suitable.*
☒	☐	For Bayesian analysis, information on the choice of priors and Markov chain Monte Carlo settings
☒	☐	For hierarchical and complex designs, identification of the appropriate level for tests and full reporting of outcomes
☐	☒	Estimates of effect sizes (e.g. Cohen's d, Pearson's r), indicating how they were calculated

Our web collection on statistics for biologists contains articles on many of the points above.

Software and code

Policy information about availability of computer code

Data collection	Histological and immunohistochemical staining images were acquired using the DS-Ri2 camera (Nikon). For immunofluorescence staining, the stained cells were imaged using a fluorescence microscope (EVOS FL Cell imaging system). For RNA-seq analysis, all procedures were performed by Macrogen using NovaSeq 6000. Transcriptome data for OA or OA-relevant conditions in various in vivo and in vitro models were obtained from the GEO database. For immunoblotting, immunoreactive protein bands were detected with iBright FL1000 (Thermo Scientific). For in vivo scanning of animals, Micro-CT Scanner (Bruker, Skyscan 1276) was used.

Data analysis

For RNA-seq, reads were preprocessed and aligned to the reference genome Mus musculus (mm10) using HISAT v2.1.0. HISAT incorporated two types of indexes for alignment: a global whole-genome index and tens of thousands of small local indexes. The reference genome sequence of mm10 and annotation data were downloaded from the UCSC genome browser (http://genome.ucsc.edu). Transcript assembly and abundance estimation were performed using StringTie v1.3.4d. The aligned reads were assembled into known, novel, and alternative splicing transcripts and the relative abundance of each transcript was quantified in read counts using StringTie v1.3.4d. For statistical analysis, genes with a read count value of zero at least in one sample were excluded. Filtered data were transformed into log2(read count+1) values and subjected to RLE normalization. Statistical significance of the differential expression was determined using nbinomWaldTest of DESeq2.
Gene ontology analysis of differentially expressed genes was performed using Enrichr (http://amp.pharm.mssm.edu/Enrichr; ver. Sep. 16th 2019). Top-ranked terms from WikiPathways, Kyoto Encyclopedia of Genes and Genomes (KEGG), BioCarta, Panther, and GO were selected by their Enrichr P values. For GSEA, gene lists of RNA sequencing data were ranked based on the fold change values (GSEA software v4.0.3).
Protein band intensity was quantified by densitometric analysis using ImageJ (v1.53e). To quantitatively analyze mRNA transcript levels, cDNA was amplified by qRT-PCR on StepOnePlus Real-Time PCR System v2.3 (Applied Biosystems). For microcomputed tomography, the three-dimensional images were constructed using NRecon software v (Bruker). For flow cytometry, BD FACSDiva software (v8.0) was used to analyze the data.
Statistical analyses were performed using IBM SPSS Statistics 25 or GraphPad Prism 9.0. All the graphs and heatmaps of log2(fold change) were plotted using GraphPad Prism 9.0.

For manuscripts utilizing custom algorithms or software that are central to the research but not yet described in published literature, software must be made available to editors and reviewers. We strongly encourage code deposition in a community repository (e.g. GitHub). See the Nature Portfolio guidelines for submitting code & software for further information.

Data

Policy information about availability of data

All manuscripts must include a data availability statement. This statement should provide the following information, where applicable:

- Accession codes, unique identifiers, or web links for publicly available datasets
- A description of any restrictions on data availability
- For clinical datasets or third party data, please ensure that the statement adheres to our policy

The original RNA-seq data generated in this study have been deposited in the GEO database under accession code GSE179535. Transcriptome data for OA or OA-relevant conditions in various in vivo and in vitro models referenced during the study are available in the GEO database (http://www.ncbi.nlm.nih.gov/geo). Transcriptomes of human OA cartilage (GSE64394 and GSE98460), IL-1β-treated cartilage explants (GSE100083), IL-1β-treated chondrocytes (GSE75181, GSE6119, and GSE104793), and cartilage from various OA animal models (DMM surgery: GSE143447; ACLT surgery: GSE110268, GSE42295, and GSE8077; MIA injection: GSE28958) were analyzed. The list of genes significantly upregulated in OA was obtained from previous studies (PMID: 26973327, 31653858). All other relevant data supporting the findings of this study are available within the article and its Supplementary Information file. Source data are provided with this paper .

Field-specific reporting

Please select the one below that is the best fit for your research. If you are not sure, read the appropriate sections before making your selection.

☒ Life sciences ☐ Behavioural & social sciences ☐ Ecological, evolutionary & environmental sciences

For a reference copy of the document with all sections, see nature.com/documents/nr-reporting-summary-flat.pdf

Life sciences study design

All studies must disclose on these points even when the disclosure is negative.

Sample size

Sample sizes were determined based on our previous experiences: PMID 24529376, 30944169, 31653858. All experiments were conducted on at least three independent biological replicates, based on the number of biological replicates required for statistical analyses. For each experiment, sample size and the number of independent biological replicates are indicated in the corresponding figure legends. Sample size required for each group for animal studies to provide sufficient power was determined based on a power analysis calculation and the design of a previous study.

Data exclusions

No exclusion criteria were included in this study.

Replication

All attempts at replication were successful. All experiments were conducted on at least three independent biological replicates.

Randomization

Cell cultures were randomly assigned to each experimental group. Animals were also randomly assigned to each experimental group, except in experiments that require specific genotypes.

Blinding

All samples were evaluated in a blinded manner. Comprehensive histological evaluation of whole-joint tissues was conducted by two orthopedic pathologists. The observers were blinded to the genotype, feeding, or surgical condition of the mice.

Reporting for specific materials, systems and methods

We require information from authors about some types of materials, experimental systems and methods used in many studies. Here, indicate whether each material, system or method listed is relevant to your study. If you are not sure if a list item applies to your research, read the appropriate section before selecting a response.

Materials & experimental systems

n/a	Involved in the study	
☐	☒	Antibodies
☒	☐	Eukaryotic cell lines
☒	☐	Palaeontology and archaeology
☐	☒	Animals and other organisms
☐	☒	Human research participants
☒	☐	Clinical data
☒	☐	Dual use research of concern

Methods

n/a	Involved in the study	
☒	☐	ChIP-seq
☐	☒	Flow cytometry
☒	☐	MRI-based neuroimaging

Antibodies

Antibodies used

Primary antibodies used for immunohistochemistry were as follows: SEPHS1 (Santa Cruz, sc-365945, clone F-6), p16INK4a (Proteintech, 10883-1-AP), MMP13 (Abcam, ab51072, clone EP1263Y), GPX1 (Abcam, ab22604), SELENOW (Rockland, 600-401-A29), 4-hydroxynonenal (Abcam, ab46545), IL-6 (Santa Cruz, sc-130326, clone 1), GROα (R&D systems, MAB453, clone 48415), ADAMTS5 (Abcam, ab41037), CTX-II (C-telopeptide of type II collagen; Novus, NBP2-59386), NITEGE (ADAMTS-cleaved aggrecan neoepitope; MD Bioproducts, 1042003, clone BC-13), HMGB1 (Abcam, ab18256), and type II collagen (Sigma Aldrich, MAB8887, clone 6B3). A previously developed MSRB1 antibody was used in this study (PMID: 14699060). Secondary antibodies used for immunohistochemistry were as follows: donkey anti-mouse IgG (H&L) conjugated with Biotin-SP (Jackson ImmunoResearch, 715-065-150; dilution 1:200), donkey anti-rabbit IgG (H&L) conjugated with Biotin-SP (Jackson ImmunoResearch, 711-065-152; dilution 1:200), and goat anti-rat IgG (H&L) conjugated with Biotin (abcam, ab6844; dilution 1:200).

Primary antibodies used for immunoblotting were as follows: SEPHS1 (Santa Cruz, sc-365945, clone F-6), GPX1 (Abcam, ab22604), SELENOW (Rockland, 600-401-A29), TXNRD1 (Santa Cruz, sc-28321, clone B-2), and actin (Santa Cruz, sc-1615, clone C-11). A previously developed MSRB1 antibody was used in this study. Secondary antibodies used for immunoblotting were as follows: goat anti-rabbit IgG (H&L) conjugated with HRP (Jackson ImmunoResearch, 111-035-003), goat anti-mouse IgG+IgM (H&L) conjugated with HRP (Jackson ImmunoResearch, 115-035-044), and donkey anti-goat IgG conjugated with HRP (Santa Cruz, sc-2020).

Primary antibodies used for immunofluorescence were as follows: γ-H2AX (Santa Cruz, sc-517348) and normal mouse IgG (Santa Cruz, sc-2025). Normal mouse IgG is an affinity purified, unconjugated isotype control immunoglobulin from mouse, recommended to use as negative controls in applications including flow cytometry, immunohistochemistry and immunofluorescence. Secondary antibody used for immunofluorescence was as rabbit anti-mouse IgG+IgM (H&L) conjugated with Alexa Fluor 488 (Jackson ImmunoResearch, 315-485-044).

Validation

Purchased or previously developed antibodies were validated by western blotting or immunohistochemistry with the use of relevant positive control, siRNA, or knockout mice. Anti-SEPHS1 (sc-365945) and anti-GPX1 (ab22604) were validated by performing western blotting and immunohistochemistry using SEPHS1 knockout mice. Anti-p16INK4a (10883-1-AP), anti-MMP13 (ab51072), anti-4-hydroxynonenal (ab46545), anti-type II collagen (MAB8887), anti-MSRB1 (previously developed antibody), anti-actin (sc-1615), and anti-γ-H2AX (sc-517348) was validated in publications from our research group (PMID: 14699060, 30944169, 31653858).

The others were validated by the manufacturer and various publications. Anti-GPX1 (ab22604) is recommended by the manufacturer for the detection of GPX1 of mouse, rat, and human origin by WB and was used by previous studies (PMID: 28263310). Anti-SELENOW (600-401-A29) is recommended by the manufacturer for the detection of SELENOW of mouse origin by WB, ELISA, and IP, and was used by previous studies (PMID: 23696641, 24392277, 24844465). Anti-IL-6 (sc-130326) is recommended for the detection of IL-6 by WB, IHC, and IF, and was used by previous studies (PMID: 29109438). Anti-GROα is recommended for the detection of GROα of mouse origin and was used by previous studies (PMID: 15187340, 23273920). Anti-ADAMTS5 (ab41037) is recommended for the detection of ADAMTS5 of mouse, rat, and human origin by WB and was used by previous studies for IHC in mouse cartilage tissue (PMID: 31253842). Anti-CTX-II (NBP2-59386) is recommended for the detection of CTX-II telopeptide of type II collagen by WB, ELISA, and IHC. Anti-NITEGE neoepitope (1042003) is recommended for the detection of NITEGE aggrecan neoepitope by WB, ELISA, and IHC, and was used by previous studies for IHC in mouse cartilage tissue (PMID: 23839930, 33452305). Anti-HMGB1 is recommended for the detection of HMGB1 of mouse, rat, and human origin by IF and WB and was used by previous studies for IHC in mouse cartilage tissue (PMID: 28436958). Anti-TXNRD1 (sc-28321) is recommended for the detection of TXNRD1 of mouse, rat, and human origin by WB, IF, and IHC, and was used by previous studies (PMID: 30799286, 31078905).

Animals and other organisms

Policy information about studies involving animals; ARRIVE guidelines recommended for reporting animal research

Laboratory animals

For primary culture of mouse articular chondrocytes, the cells were isolated from the femoral condyles and tibial plateaus of 5-day-old ICR or Sephs1-CKO littermate mice. Twelve-week-old male WT C57BL/6 or 13-week-old male Sephs1-iCKO mice were used for sham or DMM surgery. 18-month-old male Sephs1-iCKO mice or 24-month-old WT C57BL/6 were used for assessment of aging-associated OA. For knockout-based studies, respective controls (which lack Col2a1-CreERT2 in genotype) were used together. 12-week-old Selenop KO mice were also used in this study. Mice were maintained in the following conditions: temperature: 23~25'C, relative humidity: 45~65%, and light-dark cycle: 12:12 h.

Wild animals

The study did not involve wild animals.

Field-collected samples

The study did not involve samples collected from the field.

Ethics oversight

All animal experiments were approved by the SNU Institutional Animal Care and Use Committee (IACUC No. SNU-151202-6, SNU-190919-6, SNU-190910-1, SNU-191115-3).

Note that full information on the approval of the study protocol must also be provided in the manuscript.

Human research participants

Policy information about studies involving human research participants

Population characteristics	Human cartilage specimens were sourced from 22 osteoarthritis patients undergoing total knee replacement surgery. All participants are female and between 58 and 78 years of age.
Recruitment	Human OA cartilage specimens were obtained from OA patients undergoing total knee arthroplasty at SNU Boramae Medical Center. Written informed consent was obtained from all subjects before the total knee replacement surgery. There was no self-selection bias in this study.
Ethics oversight	The Institutional Review Board (IRB) of SNU Boramae Medical Center approved the collection of human biological materials (IRB No. 30-2017-48) and the IRB of Seoul National University approved the use of these materials (IRB No. E1803/003-009).

Note that full information on the approval of the study protocol must also be provided in the manuscript.

Flow Cytometry

Plots

Confirm that:

- [x] The axis labels state the marker and fluorochrome used (e.g. CD4-FITC).
- [x] The axis scales are clearly visible. Include numbers along axes only for bottom left plot of group (a 'group' is an analysis of identical markers).
- [x] All plots are contour plots with outliers or pseudocolor plots.
- [x] A numerical value for number of cells or percentage (with statistics) is provided.

Methodology

Sample preparation	For DHE staining, primary culture of mouse chondrocytes was stained with 1 µM DHE diluted in DMEM without sodium pyruvate for 15 min. For CM-H2DCFDA staining, primary culture of chondrocytes was stained with 5 µM CM-H2DCFDA diluted in DMEM without sodium pyruvate for 30 min. The stained cells were washed three times in PBS, fixed in 4% PFA for 20 min, and mounted in PBS.
Instrument	Cells were assayed by flow cytometry using FACS Canto II (BD biosciences).
Software	BD FACSDiva software (v8.0) was used to analyze the data.
Cell population abundance	At least one million cells were analyzed by flow cytometry.
Gating strategy	Cells were gated by forward scatter (FSC) and side scatter (SSC) area according to cell size and granularity to get rid of debris or large clumps. Singlet cells were selected using FSC-area (A) and FSC-height (H).

- [x] Tick this box to confirm that a figure exemplifying the gating strategy is provided in the Supplementary Information.
